# Comparative Proteomics of Phytase-transgenic Maize Seeds Indicates Environmental Influence is More Important than that of Gene Insertion

**DOI:** 10.1038/s41598-019-44748-5

**Published:** 2019-06-03

**Authors:** Yanhua Tan, Jiaming Zhang, Yong Sun, Zheng Tong, Cunzhi Peng, Lili Chang, Anping Guo, Xuchu Wang

**Affiliations:** 1 Institute of Tropical Biosciences and Biotechnology, Chinese Academy of Tropical Agricultural Sciences, Haikou, Hainan 571101 China; 2 College of Life Sciences, Key Laboratory for Ecology of Tropical Islands, Ministry of Education, Hainan Normal University, Haikou, Hainan 571158 China

**Keywords:** Biochemistry, Gene expression, Plant molecular biology, Plant physiology

## Abstract

Proteomic differences were compared between phytase-transgenic (PT) maize seeds and nontransgenic (NT) maize seeds through two-dimensional electrophoresis (2-DE) with mass spectrometry (MS). When maize was grown under field conditions, 30 differentially accumulated proteins (DAPs) were successfully identified in PT seeds (PT/NT). Clusters of Orthologous Groups (COG) functional classification of these proteins showed that the largest group was associated with posttranslational modifications. To investigate the effects of environmental factors, we further compared the seed protein profiles of the same maize planted in a greenhouse or under field conditions. There were 76 DAPs between the greenhouse- and field-grown NT maize seeds and 77 DAPs between the greenhouse- and field-grown PT maize seeds However, under the same planting conditions, there were only 43 DAPs (planted in the greenhouse) or 37 DAPs (planted in the field) between PT and NT maize seeds. The results revealed that DAPs caused by environmental factors were more common than those caused by the insertion of exogenous genes, indicating that the environment has much more important effects on the seed protein profiles. Our maize seed proteomics results also indicated that the occurrence of unintended effects is not specific to genetically modified crops (GMCs); instead, such effects often occur in traditionally bred plants. Our data may be beneficial for biosafety assessments of GMCs at the protein profile level in the future.

## Introduction

Transgenic technology has a significant influence on the development of our society^1,2^. Since the commercialization of genetically modified crops (GMCs) 20 years ago, GMCs have delivered substantial benefits to farmers and consumers at the agronomic, environmental, economic, health and social levels^3^. The area under cultivation of GMCs is increasing worldwide every year, with the area totaling more than 189.8 million hectares in 2017^4^. This increase is occurring because more consumers are willing to accept GM food^5^. However, GMCs are still at the center of intense debates and have become a source of anxiety in developing countries^6,7^, probably due to worries about their unintended, unexpected and uncontrolled negative effects^8^. To address this controversy, most people largely rely on scientific risk assessments of GMCs from the government.

To detect the potential unintended effects of GMCs, a nontargeted approach is needed to survey the plants more broadly. Omics-based global profiling, such as transcriptomics, proteomics and metabolomics, is one of the more informative and cost-efficient analytical methods and thus may be a useful technique^9,10^. Among these profiling approaches, proteomic analysis is a direct method for investigating the unintended effects at protein level. Proteins are key players in gene function and they can act as toxins, antinutrients or allergens; therefore, they are of special concern in safety assessments of GM crops. By comparing the entire proteomes of GM crops and control lines, unintended effects can be evaluated at the protein level. Currently, comparative proteomic analysis has been widely used to evaluate the unintended effects of GMCs^11–15^. Over the past 20 years, proteomics has been substantially improved in many aspects^16^. With the rapid development of proteomic technology, comparative proteomic approaches coupled directly with tandem mass spectrometry (MS) technology have been widely used to detect the unintended effects of GMCs^8^. The two-dimensional electrophoresis (2-DE) technique has been widely used in proteomics research for decades^17^. Recently, the second-generation proteomic technique iTRAQ has been widely used in comparative proteomic analyses because of its accuracy and reliability^18–20^. However, 2-DE has advantages, and many highly abundant unique proteins can be easily detected by both 2-DE and iTRAQ techniques^21^. Therefore, the 2DE and iTRAQ approaches currently represent two major techniques used in comparative proteomics^22^.

Maize is an important crop worldwide, and many maize biotechnologies have been approved; as a result, GM maize is the second largest transgenic crop in terms of planting area, reaching 59.7 million hectares in^3^. The evaluation of unintended effects in transgenic maize through proteomics is mainly performed on MON810 maize varieties because of their potential commercial value^8^. Studies have shown some differences between GMCs and their control lines, but the observed differences are not substantial^13,23–26^. In recent years, the number of stacked biotechnology events has increased, and the cultivation of transgenic crops with stacked traits has also rapidly increased^27^. Unintended effects of a stacked commercial maize hybrid were examined at the proteomics level. Compared to single-event hybrids in the same genetic background, stacking two transgenic inserts may impact the overall expression of endogenous genes and may have relevance for safety assessments^28^.

Phytase-over-expressing maize has been approved as a potential biosafe material. The transgenic maize line BVLA430101specifically expresses the 60 kDa *phy*A2 protein in its seeds^29^. We compared the proteomics of leaves between the phytase- transgenic (PT) maize and a nontransgenic (NT) isogenic variety *via* using a routine 2-DE and MS-based method^30^. Recently, we also used both 2-DE-MS/MS and iTRAQ-based methods to identify the quantitative proteomic differences between PT and NT maize seeds grown in a greenhouse^21^. Some differentially accumulated proteins (DAPs) were detected, but the proteomic patterns were not substantially different between PT maize and the NT type. In the present study, we used 2-DE with MS to compare the proteomes of PT and NT maize seeds grown in the field and under greenhouse conditions. Our results may provide more insights into the unintended effect of environmental factors on protein profiles.

## Results

### Comparison of protein profiles between field grown PT and NT maize

**Figure 1:**
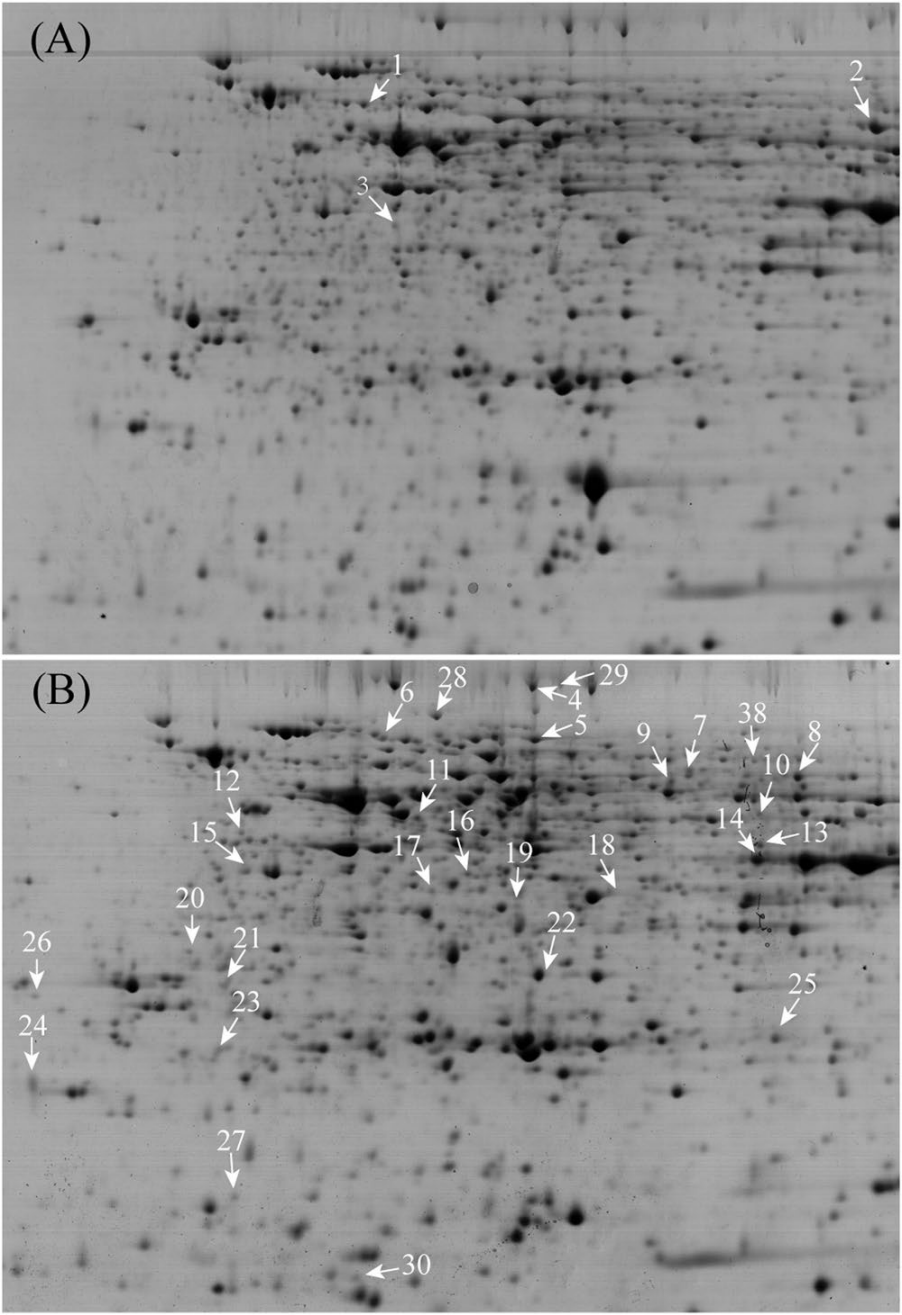
Typical 2-DE gels of total proteins from maize seeds. The identified 30 DAPs between PT and NT maize seeds, including 3 increased ones (**A**) and 27 decreased ones (**B**) in PT, are indicated with arrows in the 2-DE gels.

The 2-DE maps of total proteins from field-grown PT and NT maize seeds were obtained as previously described^30^. Analysis of the protein profiles of PT and NT maize seeds revealed a total of 1027 ± 121 spots in NT maize seed gel maps and 1228 ± 284 spots in PT maize seed gel maps (Figs 1; S1). There were approximately 1079 matched spots between NT and PT maize seed gel profiles. Only those spots showing changes of >1.5-fold or <0.67-fold and detected in all replicates were determined to be DAPs^30^. The 2-DE image analysis revealed 37 DAPs (5 higher abundance spots and 32 lower abundance spots compared with those in NT maize) between PT and NT maize seed samples grown in the field (Table S2).

### Protein identification *via* MALDI TOF/TOF MS

A total of 37 DAPs were manually excisted from colloidal Coomassie Blue (CCB)-stained 2-DE gels for MS/MS analysis and 30 protein spots were successfully identified (Fig. S2). Among these identified DAPs, 3 were up-regulated proteins, and 27 were down-regulated proteins (Fig. 1). The averaged ratio of volume% of the identified protein spots was shown in Tables 1 and S2. The database search for protein identification was based on homology to *Zea mays* proteins. If one spot was identified as containing more than one protein via MS/MS, then the protein with the highest score was chosen for further functional analysis^30^. There were 29 unique proteins in the 30 identified protein species since one protein (glucose-1-phosphate adenylyltransferase large subunit 1) was represented by two spots (Tables 1, S3). The protein that was indicated as an unknown protein was subjected to BlastP (protein-protein Blast) against the National Center for Biotechnology Information (NCBI) (http://blast.ncbi.nlm.nih.gov/Blast.cgi) to determine its identity.

**Table 1:** DAPs of maize seeds planted in the field.

Spot No^*a*^	Protein Accession No^*b*^	Protein name	Theoretical *p*I/M*r*(Da)^*c*^	Exper. *p*I/M*r*(Da)^*d*^	Coverage (%)^*e*^	Mascot score	Fold change (PT/NT)	COG classification
1	gi|195630027	Rubisco β	5.81/64.69	5.23/62.34	10	398	1.57	Post-translational modification, protein turnover, and chaperones
2	gi|189027076	glucose-1-phosphate adenylyltransferase large subunit 1	6.16/57.89	6.71/57.89	22	231	2.03	Cell wall/membrane/envelope biogenesis
3	gi|226503399	Elongation factor 2	6.00/94.89	5.27/34.66	7	254	1.52	Translation, ribosomal structure and biogenesis
4	gi|413949327	pyruvate, phosphate dikinase 2, Precursor	6.04/103.40	5.82/103.78	6	84	0.48	Energy production and conversion
5	gi|413933276	phosphoglucomutase, cytoplasmic 1	7.29/70.98	5.82/70.98	5	85	0.49	Carbohydrate transport and metabolism
6	gi|212275400	HSP 70 kDa	5.54/72.93	5.39/72.93	4	63	0.64	Post-translational modification, protein turnover, and chaperones
7	gi|226509912	ubiquitin carboxyl- terminal hydrolase 6	5.73/53.98	6.21/60.46	4	99	0.45	Post-translational modification, protein turnover, and chaperones
8	gi|189027076	glucose-1-phosphate adenylyltransferase large subunit 1	6.16/5.79	6.51/57.89	22	424	0.41	Cell wall/membrane/envelope biogenesis
9	gi|413956739	myo-inositol phosphate synthase	5.46/50.212	6.16/54.54	10	204	0.65	Lipid transport and metabolism
10	gi|162460991	indole-3-acetate beta-glucosyltransferase	5.75/50.14	6.41/45.78	4	90	0.66	Function unknown
11	gi|2500522	eIF4A	5.38/46.85	5.46/45.46	30	773	0.45	RNA processing and modification
12	gi|226529884	10-DBAT	5.03/45.91	5.0/45.34	8	171	0.64	Function unknown
13	gi|413950795	Isocitrate dehydrogenase	6.11/46.51	6.42/43.45	15	270	0.62	Energy production and conversion
14	gi|195627248	sorbitol dehydrogenase	6.27/39.53	6.40/42.16	21	411	0.50	Secondary metabolites biosynthesis, transport, and catabolism
15	gi|670397371	adenosine kinase 2-like	5.00/37.44	5.00/37.45	17	252	0.64	Nucleotide transport and metabolism
16	gi|195644252	aspartate-semialdehyde dehydrogenase	6.62/40.83	5.62/37.45	21	413	0.64	Amino acid transport and metabolism
17	gi|162464283	homocysteine S-methyltransferase 3	5.53/37.25	5.53/37.25	12	159	0.59	Amino acid transport and metabolism
18	gi|195628698	hypothetical protein	5.92/34.24	6.12/34.24	18	186	0.66	Amino acid transport and metabolism
19	gi|806638661	cysteine synthase	5.67/34.23	5.70/34.23	9	98	0.62	Amino acid transport and metabolism
20	gi|162460029	glutathione transferase41	4.85/29.09	4.83/32.09	19	153	0.61	Post-translational modification, protein turnover, and chaperones
21	gi|226495167	desiccation-related protein PCC13-62 precursor	4.82/34.24	4.96/29.67	9	93	0.66	Function unknown
22	gi|226493460	TSJT1	5.23/25.05	5.93/28.78	8	125	0.65	Function unknown
23	gi|226499536	NADH-ubiquinone oxidoreductase 23 kDa subunit	5.24/26.09	4.94/26.45	17	210	0.62	Energy production and conversion
24	gi|32330695	SKP1/ASK1 protein	4.48/19.20	4.42/23.31	19	301	0.65	Post-translational modification, protein turnover, and chaperones
25	gi|195636212	rhicadhesin receptor precursor	6.58/22.95	6.64/23.23	30	385	0.56	Function unknown
26	gi|195640298	glycine-rich RNA-binding protein 7	4.87/25.16	4.41/28.12	8	86	0.43	RNA processing and modification
27	gi|162457809	ubiquitin-conjugating enzyme protein E2	5.04/19.07	4.56/19.07	21	85	0.62	Post-translational modification, protein turnover, and chaperones
28	gi|222623975	hypothetical protein OsJ_08986	5.82/88.30	5.52/88.30	3	81	0.53	Post-translational modification, protein turnover, and chaperones
29	gi|413949328	pyruvate, phosphate dikinase 3, Precursor	6.04/103.78	5.78/103.78	5	103	0.38	Energy production and conversion
30	gi|48374986	hypothetical protein Z477F24.14	4.94/15.60	5.28/16.53	9	66	0.55	Carbohydrate transport and metabolism

Note:

^a^Assigned spot numbers as indicated in Fig. 1.

^b^Database accession numbers according to NCBI.

^c,d^The theoretical (c) and experimental (d) values of molecular weight (M*r*., kDa) and *p*I for the identified proteins.

^e^Percent values of coverage (%) of the matched peptides in the whole protein sequence.

**Figure 2:**
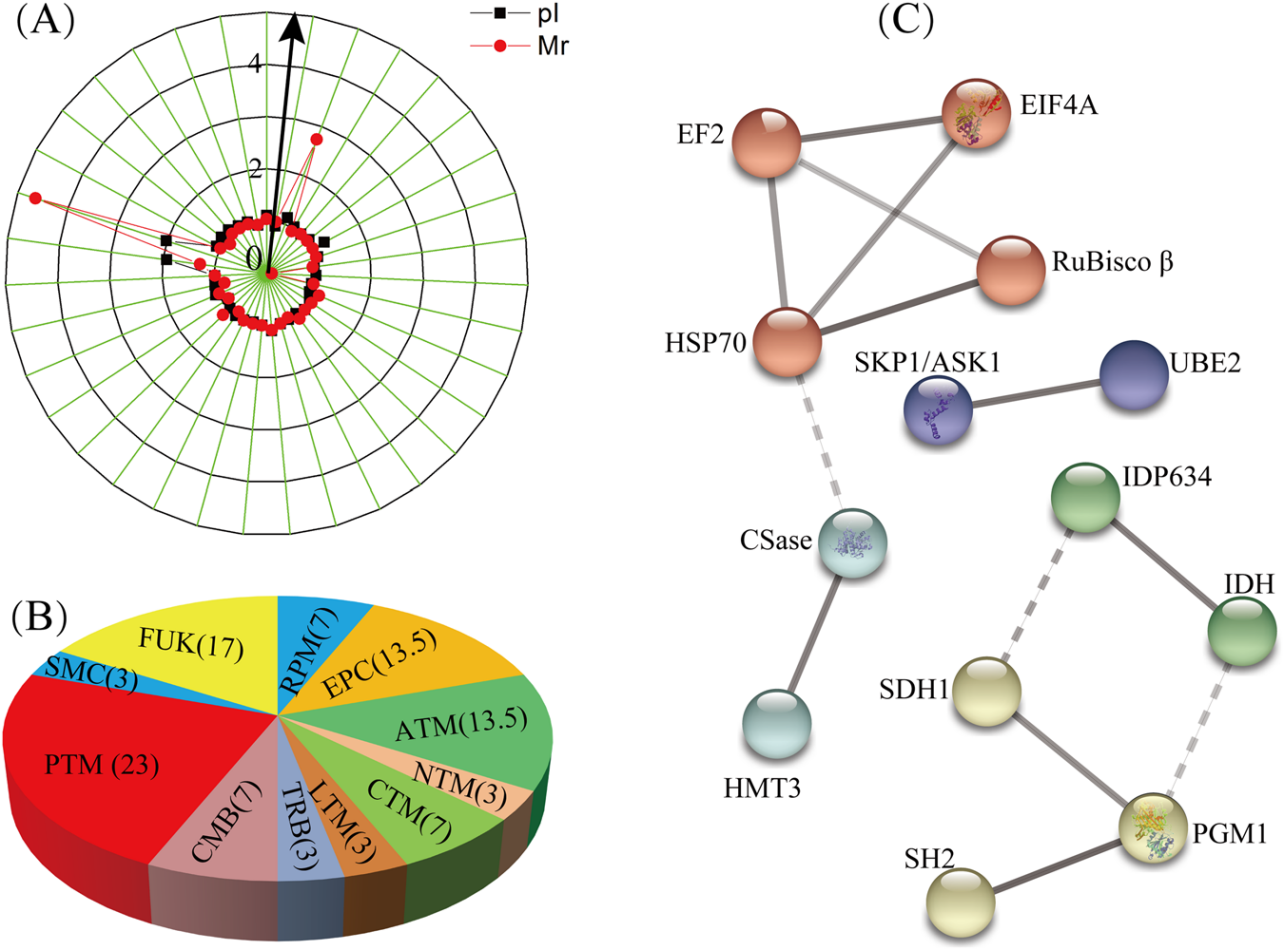
Classification and protein-protein interaction analysis of the identified DAPs. The theoretical and experimental ratios of the molecular mass (M*r*) and isoelectric points (*p*I) of the 30 identified DAPs are presented in the radial chart (**A**). Functional classification was produced by COG, and the results are provided as the percent proportion (%) of each functional category in all identified DAPs (**B**). The abbreviations in the figures are as follows: CTM, carbohydrate transport and metabolism; RPM, RNA processing and modification; PTM, posttranslational modification, protein turnover, chaperones; TRB, translation, ribosomal structure and biogenesis; LTM, lipid transport and metabolism; CMB, cell wall/membrane/envelope biogenesis; EPC, energy production and conversion; ATM, amino acid transport and metabolism; NTM, nucleotide transport and metabolism; SMC, secondary metabolites biosynthesis, transport, and catabolism; FUK, function unknown. The hidden disconnected nodes in the protein-protein interaction networks are shown in the five tightly connected clusters after MCL clustering (**C**).

A radial chart was used to evaluate the quality of the identified protein spots. The theoretical ratios and experimental ratios of the molecular mass (M*r*) were presented in the radial chart as the radial axis labels, and the theoretical ratios and experimental ratios of the isoelectric point (*p*I) are presented as the annular radial axis labels (Fig. 2A). Approximately 91% of the identified proteins exhibited a relative M*r* ratio in the range of 1.0 ± 0.2, and 94.3% of the identified proteins exhibited a relative *p*I ratio in the range of 1.0 ± 0.2, which suggested that most identified proteins’ experimental M*r* and *p*I values were similar to their theoretical values.

### Bioinformatics analysis of the identified DAPs

The identified DAPs were grouped according to their main biological activities as defined by the functional catalogue of Clusters of Orthologous Groups (COG) of proteins. COG functional analysis classified 25 of the identified proteins into 10 major categories, among which “posttranslational modification, protein turnover, chaperones” was the largest group (group PTM, containing 23% of the DAPs), followed by “energy production and conversion” (group EPC, 13.5% DAPs), and “Amino acid transport and metabolism” (group ATM, 13.5% DAPs). The remaining categories were “RNA processing and modification” (group RPM, 7% DAPs), “carbohydrate transport and metabolism” (group CTM, 7% DAPs), “cell wall/membrane/envelope biogenesis” (group CMB, 7% DAPs), “nucleotide transport and metabolism” (group NTM, 3% DAPs), “secondary metabolites biosynthesis, transport, and catabolism” (group SMC, 3% DAPs), lipid transport and metabolism(group LTM, 3% DAPs), and “translation, ribosomal structure and biogenesis” (group TRB, 3% DAPs); 5 proteins could not be classified through COG classification (Fig. 2B, Table 1).

To predict protein-protein interaction networks, the 29 identified unique proteins were subjected to STRING (v10.5) analysis online (http://string-db.org) with high confidence. Among these proteins, 13 were involved in protein-protein interactions with 3 up-regulated and 10 down-regulated proteins. Hided disconnected nodes in the network, there were five tightly connected clusters after MCL clustering (Fig. 2C). There were 4 proteins in the red cluster, including HSP70, EIF4A, EF2, and RuBisco β. HSP70 and EF2 were found to be the most interactive proteins in these interaction networks, associating with three other proteins, followed by the yellow cluster, with 3 proteins. The 3 remaining clusters contained two proteins that interacted with each other. Among these proteins, four interacting proteins were mainly related to “post-translational modification, protein turnover, and chaperones”, while three interacting proteins were related to “energy production and conversion” among the COG categories.

**Figure 3:**
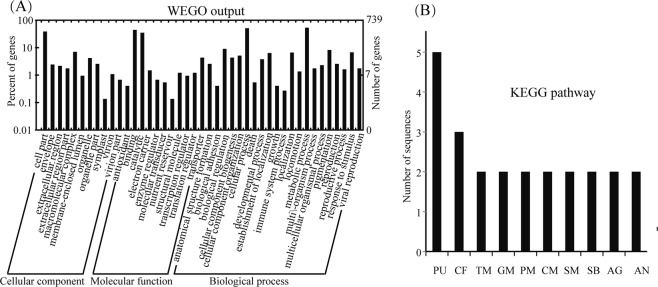
GO annotation and pathway analysis of the identified DAPs. The identified 30 DAPs between the PT and NT maize seeds planted in the field were subjected to GO (**A**) and KEGG (**B**) analyses. The abbreviations for the KEGG pathways are as follows: PU, purine metabolism; CF, carbon fixation; TM, thiamine metabolism; GM, glutathione metabolism; PM, pyruvate metabolism; CM, cysteine and methionine metabolism; SM, starch and sucrose metabolism; SB, streptomycin biosynthesis; AG, alanine, aspartate and glutamate metabolism; AN, amino sugar and nucleotide sugar metabolism.

To confirm the significantly enriched Gene Ontology (GO) functional groups of the identified DAPs in cellular component, biological process, and molecular function categories, GO annotation was further conducted through an online search using WEGO software (http://wego.genomics.org.cn/cgi-bin/wego/index.pl). GO information was obtained with BLAST2GO. The results showed that 30 proteins were successfully mapped with GO annotations, which were classified into three ontologies containing 43 functional groups (Fig. 3A). At the cellular level, 11 GO terms were obtained, including the cellular component category (GO: 00044464), which contained 38.7% of the proteins. For the molecular function ontology, 11 GO terms were found, and the major functional groups were binding functional groups (GO: 0005488), containing 44.7% of the proteins, and catalytic activity (GO: 0003824), containing 35% of the proteins. In the biological process, 21 GO terms were assigned. The major functional group of the proteins was involved in metabolic process (GO: 0008152), including 53.6% of the proteins, followed by cellular processes (GO: 0009987) with 51.2% of the proteins.

A Kyoto Encyclopedia of Genes and Genomes (KEGG) pathway analysis of the identified DAPs was performed using the BLAST2GO 4.0 program to investigate their biological functions. The results showed that a total of 18 proteins (58%) were mapped to 28 pathways in the KEGG database. The most represented pathway was “purine metabolism”, which contained five sequences (spots 3, 5, 11, 15 and 27). The other major pathway was “carbon fixation pathways” which contained three sequences (spots 4, 13 and 30). Two proteins were involved in each of the following pathways: “thiamine metabolism”, “starch and sucrose metabolism”, “glutathione metabolism”, “pyruvate metabolism”, “cysteine and methionine metabolism”, “streptomycin biosynthesis”, “alanine, aspartate and glutamate metabolism”, and “amino sugar and nucleotide sugar metabolism”. The remaining pathways contained only one protein sequence (Fig. 3B, Table S4).

### Comparison of the protein accumulation and gene expression patterns

**Figure 4:**
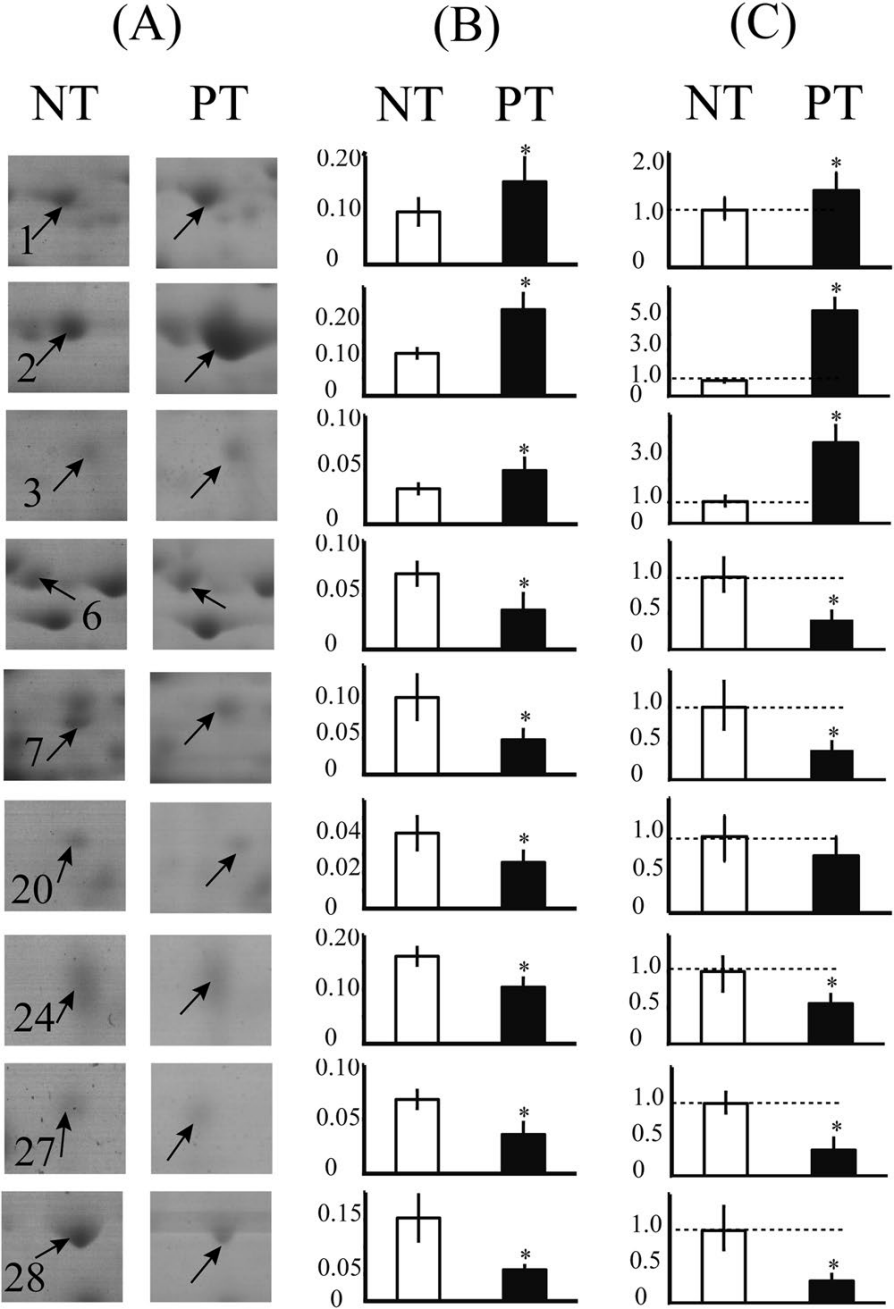
Comparison of the changes in the identified DAPs at protein abundance and gene expression levels. The selected protein spots in the 2-DE gel profiles of NT and PT maize seeds are highlighted (**A**). The mean abundance values (Vol%) of these spots were calculated (**B**). Results of qRT-PCR analysis of the corresponding gene expression patterns of the identified proteins are shown in column (**C**). The gray dotted line in each qRT-PCR bar chart represents a 1.0 ratio value. Error bars represent the standard deviation (SD) among three replicates. The comparison showed that almost all the examined genes and proteins exhibited a similar pattern in the maize seeds.

We selected ten identified proteins for *q*RT-PCR analysis to validate the expression patterns of their corresponding genes. To obtain the PT/NT fold-change ratios, the transcript level of the NT maize template was set to 1.0. The changes in the protein accumulation and mRNA expression levels of the selected identified proteins are shown in Fig. 4. Most of the proteins exhibited similar changes at the translational and transcriptional levels; only one down-regulated protein (glutathione transferase 41, spot 20) showed no difference at the transcriptional level. Such inconsistency between the patterns of change in protein accumulation and mRNA expression levels was described in our previous studies^21,30^; this phenomenon probably resulted from the presence of various posttranslational modifications^31^.

### Comparison of protein profiles in maize seeds from different environments

**Figure 5:**
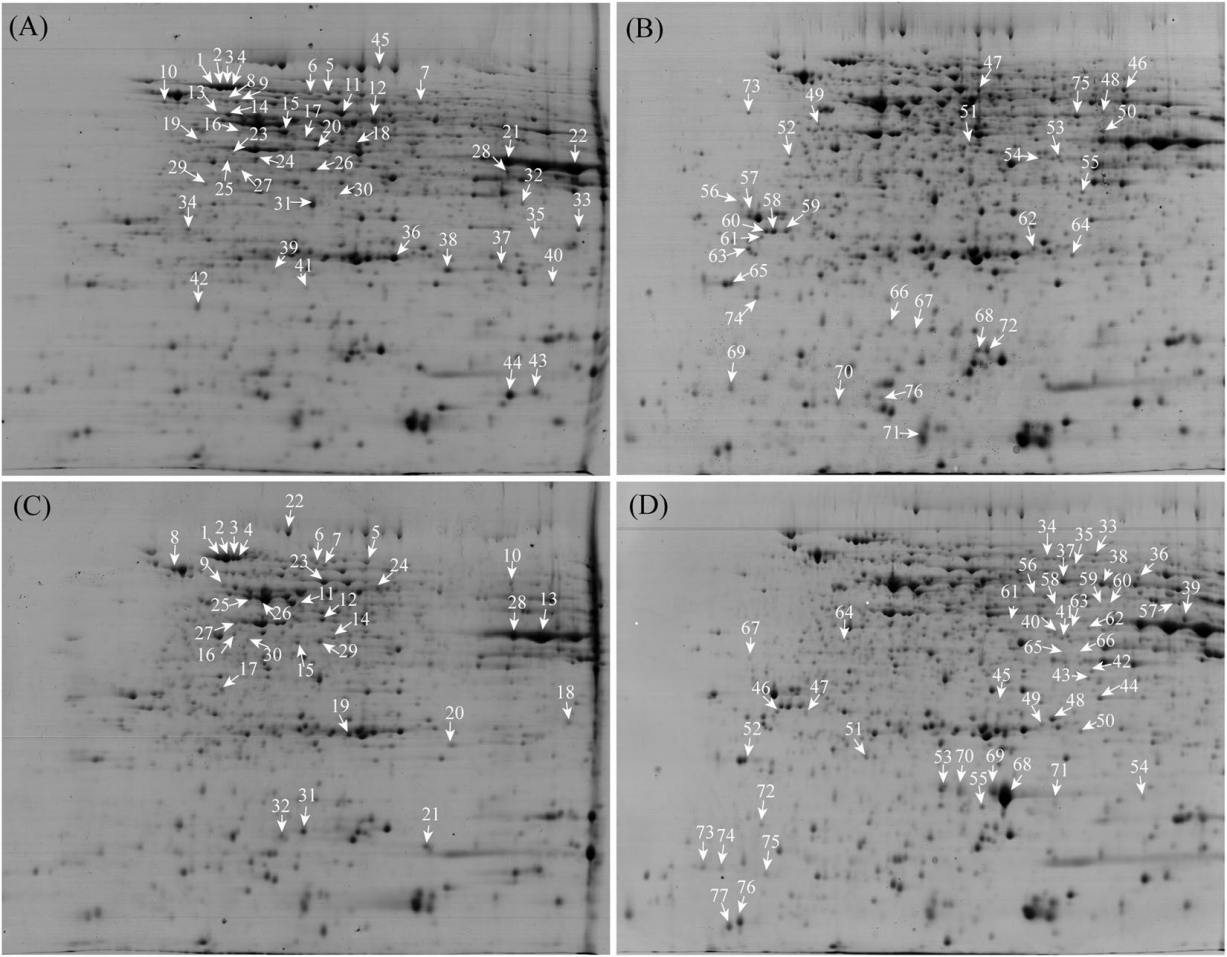
Typical 2-DE gels of the proteins from maize seeds under different growth environments. The proteins from seeds of NT plants grown under greenhouse (**A**) and field (**B**) conditions, as well as PT plants in the greenhouse (**C**) and the field (**D**), were subjected to 2-DE, and the DAPs in typical 2-DE gels are highlighted. Arrows indicate the protein spots with an increased abundance in each sample.

We identified quantitative differences in the protein profiles between greenhouse-planted PT and NT maize seeds using both the traditional 2-DE and the newly developed high-throughput iTRAQ-based approaches^21^. Then we compared the protein profiles between field-planted PT and NT maize seeds using traditional 2-DE approaches. To analyze the effects of different planting environments on the PT maize seeds and the control, we further compared the 2-DE gel profiles of maize seeds planted in the field or in a greenhouse (Figs 5, S1, Table 2). The protein spots with changes >1.5-fold were termed as DAPs. There were 76 DEPs between the NT maize seeds grown in two different environments, including 45 up-regulated protein spots in the greenhouse and 31 up-regulated protein spots in the field (Fig. 5A,B, Table S5). Seventy-seven DEPs were detected in the PT maize seeds, with 32 up-regulated protein spots in the greenhouse and 45 up-regulated ones in the field (Fig. 5C,D, Table S6). However, as mentioned above, after comparing the 2-DE profiles of PT and NT maize seeds in the same planting environment, there were only 43 DAPs (PT/NT, planted in the greenhouse)^21^ or 37 DAPs (PT/NT, planted in the field). These results demonstrated that the growth environment was more important than the gene modification itself for the protein profiles in maize seeds.

**Table 2:** Comparison of the DAPs of maize seeds planted under different conditions.

2-DE maps in different planting environments	Matched spots	DAPs	Up-regulated spots	down-regulated spots
PT/NT (greenhouse)	990	43	18	25
PT/NT (field)	1079	37	5	32
NT/NT (greenhouse/field)	690	76	45	31
PT/PT (greenhouse/field)	1079	77	32	45

## Discussion

### Many DAPs in the field-grown maize seeds were posttranslational modification-related chaperone proteins

The 30 identified DAPs were obtained between PT and NT maize seeds, which were collected from the field. COG functional classification showed that the largest group (23% of the DAPs) was associated with “posttranslational modification, protein turnover, chaperones”, such as HSP70, ubiquitin carboxyl-terminal hydrolase, glutathione transferase, and ubiquitin-conjugating enzyme. Under field conditions, plants are vulnerable to various stresses, such as drought, disease and insect pests. Posttranslational modification proteins may play important roles in response of abiotic stresses^32^. As a chaperone protein, HSP70 promotes the degradation of aberrant proteins, prevents the aggregation of denatured proteins and promotes proper folding of denatured proteins^33,34^. Ubiquitination is an important process in all eukaryotic cells, and the ubiquitin proteasome pathway participates in all aspects of the regulation of eukaryotic cells due to the degradation of proteins in such cells^35,36^. Ubiquitin-conjugating enzyme E2 can catalyze ubiquitin substrate transfer to protein hydrolysis^37^.

### Environmental influence is more important than gene insertion

In evaluating the unintended effects in GMCs, an important factor to consider is the impact of environmental conditions during maize planting^38^. We compared the proteomics of PT maize seeds and a control planted in a greenhouse to eliminate variation related to the genome alteration^21^. In contrast, comparing the proteomic profiles of the same variety (NT/NT, PT/PT) grown under different environmental conditions enabled the elimination of any variation related to the environmental effects on maize seed proteomic profiles^26^.

In a comparison of the seed proteome profiles of the same variety grown under different environmental conditions, e.g., in a greenhouse or the field, DAPs would be related to the environmental impact. The genomes of NT or PT maize seeds were not different between the greenhouse and field. The 2-DE gel maps of NT maize seeds revealed 76 DAPs between the greenhouse and field-planted seeds, and similarly, there were 77 DAPs in PT maize seeds between greenhouse and field planted samples. However, under the same growth conditions, there were only 43 DAPs (greenhouse) or 37 DAPs (field) when PT maize was compared with NT maize. These data revealed that the insertion of exogenous genes can lead to plant genomic changes causing DAPs, but the influence of the environment on protein profiles (numbers of DAPs) is stronger than the influence of exogenous genes. We think that environmental factors have more important effects than exogenous gene insertion on seed protein profiles. In addition, comparative proteomics of NT maize seeds planted in a greenhouse vs. in the field also revealed that the occurrence of unintended effects is not specific to GM crops. This is a common inherent phenomenon, as it often occurs in the traditional breeding of crops. Environmental impacts on crops are much stronger than those of gene insertion, which is consistent with a previous report^26,39^. Previous observations also indicated that transgenes have very limited unintended effects, while large differences were observed between lines produced by conventional breeding^40–43^.

To clearly understand whether PT maize causes unintended effects, we systematically compared the proteomics of seedling leaves and seeds between PT and NT maize grown under control conditions^21,30^. We detected insubstantial differences between the seeds of PT maize and those of NT maize. In this study, we further compared the proteomes of PT and NT maize seeds planted in the field condition, and 30 DAPs were successfully identified in these samples. COG functional classification showed that the largest group was associated with “posttranslational modification, protein turnover, chaperones”. In addition, we compared the seed proteome profiles of the same maize species but grown in different locations. Our results revealed that the number of DAPs caused by the environment was much greater than that caused by the insertion of exogenous genes. Thus, the environment had more important effects on seed protein profiles than exogenous gene insertion, as it. The occurrence of unintended effects is not specific to GM crops, and it often occurs in traditional breeding. Our comparative proteomics techniques serve as an exploratory method to determine the safety of GM maize seeds. In addition, in this study, a proteomic comparison of maize seeds was carried out for only one season of field planting. However, the proteome is highly dynamic and can be changed by the cell cycle, environmental influences, and tissue/cell types^44^. Therefore, the proteomes in long-term- and multi-season-planted maize seeds need to be further compared. In conclusion, the proteomics data of PT maize seeds provided much more information and will be beneficial for the biosafety assessment of PT maize in the future.

## Materials and Methods

### Plant materials and growth conditions

The phytase-transgenic maize variety is 10TPY006 (PT maize), and the corresponding near-isogenic variety is the conventional hybrid LIYU16 (NT maize). PT and NT maize seeds were provided by Beijing Origin Seed Technology, Inc. The genetic background of the materials was as previously described^30^. First, conventional maize (LIYU91158 and LIYU953) was crossed with the *phy*A2 transgenic maize line BVLA430101, and a *phy*A2-insertion event was introduced into the LIYU91158 and LIYU953 backgrounds. Then, the LIYU91158 and LIYU953 transgenic lines were backcrossed six times to their recurrent parents to minimize genetic background mixing, and two self-pollinations were performed to obtain homozygous plants (OSL931 and OSL930) of each inbred line. Because its DNA was similar to that of LIYU16, the GM line LIYU006 was further derived by crossing OSL931 and OSL930. In the same manner, the NT line of LIYU16 (used as a non-GM control) was derived by crossing the LIYU91158 and LIYU953 inbred lines as described in our previous study^30^. Materials were planted at the experimental base of the Institute of Tropical Biosciences and Biotechnology (E: 110°45′42″; N: 19°32′18″). These PT and NT seeds were planted side-by-side in the field, and each line was planted in three microplots to represent three replicates. These maize seeds were planted in the same experimental sites as those grown in a greenhouse^21^. After sowing, the plants were treated according to local agricultural practices. Ears of each microplot were harvested at the same time on the same day when physiologically mature and immediately stored at −80 °C for further study.

### Protein extraction

For comparative proteomic analysis, central seeds of each ear were ground into fine powders in liquid nitrogen using a mortar and pestle. Semiquantitative RT-PCR and western blotting analysis were conducted to detect the expression of exogenous genes and the accumulation of target proteins as described previously^21,30^.

Three biological replicates of PT and NT seed proteins were extracted using a modified Borax/PVPP/Phenol (BPP) protein extraction method as described previously^21,45^. Approximately 3 g of frozen maize seed fine powders were resuspended in precooled extraction buffer. After added an equal volume Tris-saturated phenol (pH 8.0), the mixtures were centrifuged. Then the upper phase was transferred into a new centrifuge tube and clarified twice. After adding ammonium sulfate saturated-methanol and protein precipitates were obtained. The proteins were quantified according to the Bradford method for the following experiments or were stored at −20 °C.

### 2-DE

2-DE was performed on an Ettan IPGphor isoelectric focusing system according to the manufacturer’s instructions (2-DE Manual, GE Healthcare, Uppsala, Sweden). The 24 cm IPG strips (immobilized pH gradient) with a linear pH gradient of 4–7 (GE Healthcare) were used, approximately 1,300 µg protein samples were loaded on, and 12.5% sodium dodecyl sulfate (SDS) polyacrylamide gels were used for SDS- polyacrylamide gel electrophoresis (SDS-PAGE). Each protein extracts were performed on 2-DE gels in triplicate for technical replicates. The experimental procedures were as previously described^30^.

Gels were stained using a GAP staining method^46^ and scanned with the ImageMaster Labscan V3.0 (GE Healthcare). Image analysis was conducted using a ImageMaster 2D Platinum software package (GE Healthcare). Only the spots that were present in all replicate gels and shown a Student’s *t* test p-value < 0.05 and a relative change in quantity of at least 1.5-fold in their quantity, were considered as DAPs for further analysis^30^.

### Protein identification in 2-DE Gels *via* MALDI TOF MS

DAPs were manually excised from 2-DE gels, washed with MilliQ water, and then destained using a destaining solution containing 50 mM NH_4_HCO_3_ and 50% acetonitrile (ACN). After air dried, in-gel digestion with bovine trypsin (Trypsin, Roche, Cat. 11418025001) was performed as previously described^47^.

The digested protein peptides were detected for peptide map fingerprinting (PMF) by using an AB SCIEX matrix-assisted laser desorption/ionization time-of-flight (MALDI TOF) 5800 system (AB SCIEX, Shanghai, China) equipped with neodymium and a laser wavelength of 349 nm. Mass spectra were obtained as previously described^48^ and searched against the *Zea mays* amino acid sequence database (including 87,603 sequences) using MASCOT software in-house for protein identification. The search parameters were set as described^30^. If peptides matched to multiple proteins, the protein with the highest score was selected for bioinformatics analysis. For unknown proteins, a BLAST search was performed in NCBI (http://www.ncbi.nlm) to identify homologous proteins.

### Bioinformatics analysis

Functional annotations of the identified DAPs were performed. COG analysis of DAPs was conducted for functional classification through an online search (http://eggnogdb.embl.de/). GO classification was carried out online using WEGO software according to GO terms as described (http://wego.genomics.org.cn)^49^. In addition, KEGG pathways were analyzed to predict the main reaction networks in which DAPs were involved in using Blast2GO 4.0 software. Finally, protein-protein interactions were analyzed using the STRING database (version 10.5) online (http://string-db.org) and network was clustered to a specified “MCL inflation parameter”.

### qRT-PCR analysis

Total RNA was isolated from maize seeds with TRIzol reagent (CWBIO, Beijing, China), and cDNA was generated with a reverse transcriptase kit (TaKaRa, Tokyo, Japan) for quantitative real-time RT-PCR. Approximately 20 μL of mixed solution was prepared for qRT-PCR reaction using SYBR Green PCR Master Mix (TaKaRa, Tokyo, Japan), and the reactions were performed on an Mx3005P sequence detection system according to the manufacturer’s instructions. The maize endogenous gene *zSSIIb* was used as an internal control to normalize the amount of template cDNA. qRT-PCR primer pairs were designed with Primer 5.0 software (Table S1). Data were analyzed with MxPro software (version 4.10).

## Supplementary information

Supplementary Figure S1, Supplementary Figure S2

Table S1, Table S2,Table S3, Table S4, Table S5, Table S6

## References

[1] Christou P (2013). Plant genetic engineering and agricultural biotechnology 1983–2013. Trends Biotechnol..

[2] Ahmad N, Mukhtar Z (2017). Genetic manipulations in crops: Issues and opportunities. Genomics..

[3] ISAAA. Global status of commercialized biotech/GM crops: 2016. *ISAAA Brief*. 52. ISAAA: Ithaca, NY (2017).

[4] ISAAA. Global Status of Commercialized Biotech/GM Crops in 2017: Biotech Crop Adoption Surges as Economic Benefits Accumulate in 22 Years. *ISAAA Brief*. 53. ISAAA: Ithaca, NY (2018).

[5] De Steur H, Wesana J, Blancquaert D, Van Der Straeten D, Gellynck X (2017). The socioeconomics of genetically modified biofortified crops: A systematic review and meta-analysis. Ann. N. Y. Acad. Sci..

[6] Hartley S, Gillund F, van Hove L, Wickson F (2016). Essential features of responsible governance of agricultural biotechnology. PLoS Biol..

[7] Azadi Hossein, Taube Friedhelm, Taheri Fatemeh (2017). Co-existence of GM, conventional and organic crops in developing countries: Main debates and concerns. Critical Reviews in Food Science and Nutrition.

[8] Gong CY, Wang T (2013). Proteomic evaluation of genetically modified crops: Current status and challenges. Front. Plant Sci..

[9] Kuiper HA, Kleter GA, Noteborn HPJM, Kok EJ (2001). Assessment of the food safety issues related to genetically modified foods. Plant J..

[10] Ladics GS (2015). Genetic basis and detection of unintended effects in genetically modified crop plants. Transgenic Res..

[11] Scossa F (2008). Comparative proteomic and transcriptional profiling of a bread wheat cultivar and its derived transgenic line overexpressing a low molecular weight glutenin subunit gene in the endosperm. Proteomics..

[12] Khalf M (2010). Tubers from potato lines expressing a tomato Kunitz protease inhibitor are substantially equivalent to parental and transgenic controls. Plant Biotechnol. J..

[13] Coll A, Nadal A, Rossignol M, Puigdomenech P, Pla M (2011). Proteomic analysis of MON810 and comparable non-GM maize varieties grown in agricultural fields. Transgenic Res..

[14] Barbosa H, Arruda S, Azevedo R, Arruda M (2012). New insights on proteomics of transgenic soybean seeds: Evaluation of differential expressions of enzymes and proteins. Anal. Bioanal. Chem..

[15] Gong CY, Li Q, Yu HT, Wang Z, Wang T (2012). Proteomics insight into the biological safety of transgenic modification of rice as compared with conventional genetic breeding and spontaneous genotypic variation. J. Proteome Res..

[16] Lenz C, Dihazi H (2016). Introduction to proteomics technologies. Methods. Mol Biol..

[17] Rogowska-Wrzesinska A, Le Bihan MC, Thaysen-Andersen M, Roepstorff P (2013). 2D gels still have a niche in proteomics. J. Proteomics..

[18] Fukao Y (2011). iTRAQ analysis reveals mechanisms of growth defects due to excess Zinc in arabidopsis. Plant Physiol..

[19] Ghochani BBFNM, Gilany K (2011). Proteomics a key tool for a better understanding of endometriosis: a mini- review. J. Paramedical Sci..

[20] Zieske LR (2006). A perspective on the use of iTRAQ TM reagent technology for protein complex and profiling studies. J. Exp. Bot..

[21] Tan YH (2017). Proteomic analysis of phytase transgenic and non-transgenic maize seeds. Scientific Reports..

[22] Wu X, Wang W (2016). Increasing confidence of proteomics data regarding the identification of stress-responsive proteins in crop plants. Front. Plant Sci..

[23] Albo AG (2007). Proteomic analysis of a genetically modified maize flour carrying Cry1Ab gene and comparison to the corresponding wild-type. Maydica..

[24] Balsamo GM, Cangahuala-Inocente GC, Bertoldo JB, Terenzi H, Arisi AC (2011). Proteomic analysis of four Brazilian MON810 maize varieties and their four non-genetically-modified isogenic varieties. J. Agric. Food Chem..

[25] Vidal N, Barbosa H, Jacob S, Arruda M (2015). Comparative study of transgenic and non-transgenic maize (*Zea mays*) flours commercialized in Brazil, focussing on proteomic analyses. Food Chem..

[26] Zolla L, Rinalducci S, Antonioli P, Righetti PG (2008). Proteomics as a complementary tool for identifying unintended side effects occurring in transgenic maize seeds as a result of genetic modifications. J. Proteome Res..

[27] Kamle M, Kumar P, Patra JK, Bajpai VK (2017). Current perspectives on genetically modified crops and detection methods. 3 Biotech..

[28] Agapito-Tenfen S (2014). Effect of stacking insecticidal cry and herbicide tolerance epsps transgenes on transgenic maize proteome. BMC Plant Biol..

[29] Chen R (2008). Transgenic maize plants expressing a fungal phytase gene. Transgenic Res..

[30] Tan YH (2016). Comparative proteomics of leaves from phytase-transgenic maize and its non-transgenic isogenic variety. Front. Plant Sci..

[31] Wang X (2016). Itraq-based quantitative proteomic analysis reveals new metabolic pathways responding to chilling stress in maize seedlings. J. Proteomics..

[32] Mazzucotelli E (2008). Abiotic stress response in plants: When post-transcriptional and post-translational regulations control transcription. Plant Science..

[33] Parsell DA, Lindquist S (1993). The function of heat-shock proteins in stress tolerance: Degradation and reactivation of damaged proteins. Genetics..

[34] Wang W, Vinocur B, Shoseyov O, Altman A (2004). Role of plant heat-shock proteins and molecular chaperones in the abiotic stress response. Trends Plant Sci..

[35] Callis J, Vierstra RD (2000). Protein degradation in signaling. Curr. Opin. Plant Biol..

[36] Hershko A, Ciechanover A (1992). The ubiquitin system for protein degradation. Annu. Rev. Biochem..

[37] Zhou GA, Chang RZ, Qiu LJ (2010). Overexpression of soybean ubiquitin-conjugating enzyme gene GmUBC2 confers enhanced drought and salt tolerance through modulating abiotic stress-responsive gene expression in arabidopsis. Plant Molecular Biology..

[38] Consoli, L. & Damerval, C. Quantification of individual zein isoforms resolved by two-dimensional electrophoresis: Genetic variability in 45 maize inbred lines. *Electrophoresis*. **22**, 2983–2989, 10.1002/1522-2683 (200108)22:14<2983::AID-ELPS2983>3.0.CO;2-# (2001). 10.1002/1522-2683(200108)22:14<2983::AID-ELPS2983>3.0.CO;2-# 11565792

[39] Frank T, Röhlig RM, Davies HV, Barros E, Engel KH (2012). Metabolite profiling of maize kernels–genetic modification versus environmental influence. J. Agric. Food Chem..

[40] Catchpole GS (2005). Hierarchical metabolomics demonstrates substantial compositional similarity between genetically modified and conventional potato crops. Proceedings of the National Academy of Sciences of the United States of America..

[41] Ioset J (2007). Flavonoid profiling among wild type and related GM wheat varieties. Plant Molecular Biology..

[42] Lehesranta SJ (2005). Comparison of tuber proteomes of potato varieties, landraces, and genetically modified lines. Plant Physiology..

[43] Marcela BM (2006). Transgenesis has less impact on the transcriptome of wheat grain than conventional breeding. Plant Biotechnol J..

[44] Pastorello EA (2000). The maize major allergen, which is responsible for food-induced allergic reactions, is a lipid transfer protein. J. Allergy and Clinical Immunology..

[45] Wang X (2007). A protein extraction method compatible with proteomic analysis for euhalophyte salicornia europaea. Electrophoresis..

[46] Wang X (2012). Systematic comparison of technical details in CBB methods and development of a sensitive GAP stain for comparative proteomic analysis. Electrophoresis..

[47] Wang X (2009). Comparative proteomic analysis of differentially expressed proteins in shoots of salicornia europaea under different salinity. J. Proteome Res..

[48] Yi X (2014). Quantitative proteomics of sesuvium portulacastrum leaves revealed that ion transportation by V-ATPase and sugar accumulation in chloroplast played crucial roles in halophyte salt tolerance. J. Proteomics..

[49] Ye J (2006). Wego: A web tool for plotting go annotations. Nucleic Acids Res..

