# Supplementary material for: Comparative Proteomics of Phytase-transgenic Maize Seeds Indicates Environmental Influence is More Important than that of Gene Insertion
Source: Sci Rep. 2019 Jun 3;9:8219. doi: 10.1038/s41598-019-44748-5 (PMC6547748; doi:10.1038/s41598-019-44748-5)
Supplement: Supplementary file 1 — Supplementary Figure S1, Supplementary Figure S2 [file 41598_2019_44748_MOESM1_ESM.pdf]

Comparative Proteomics of Phytase-transgenic Maize Seed Indicates Environmental Influence is More Important than that of Gene Insertion

Authors: Yanhua Tan, Jiaming Zhang, Yong Sun, Zheng Tong, Cunzhi Peng, Lili Chang, Anping Guo, Xuchu Wang

Supplementary Figure S1: 2D gels of maize seed's proteins in different growth environments.

Supplementary Figure S2: Supplemental spectra and MALDI TOF/TOF MS/MS identification of the differentially expressed proteins.

Supplementary Figure S1: 2D gels of maize seed's proteins in different growth environments.

(1): 3 repeated 2D gels of NT maize seeds grown in greenhouse.

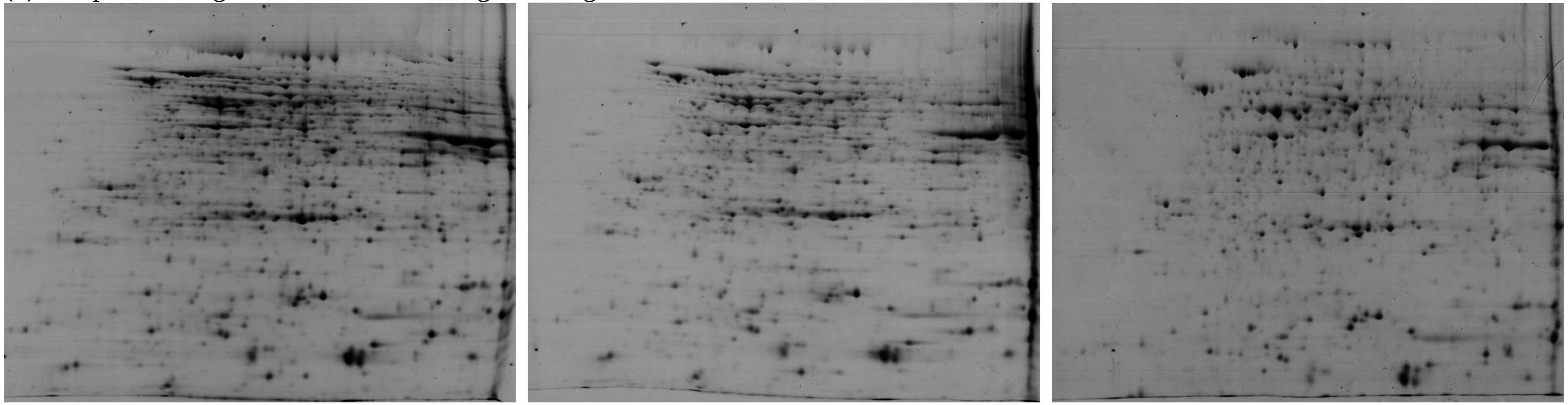

(2): 3 repeated 2D gels of NT maize seeds grown in field.

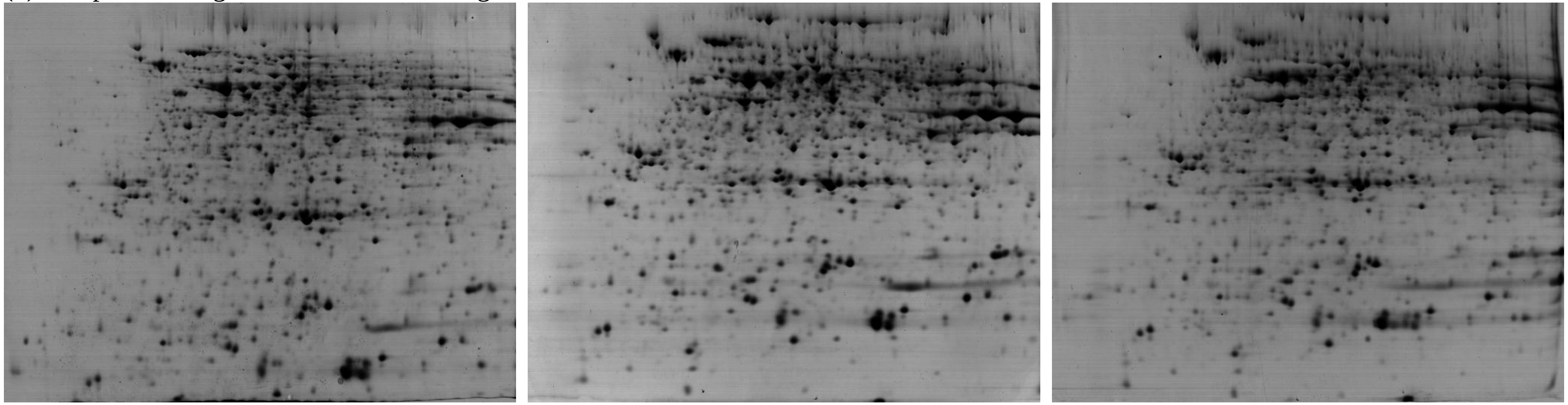

(3): 3 repeated 2D gels of PT maize seeds grown in greenhouse.

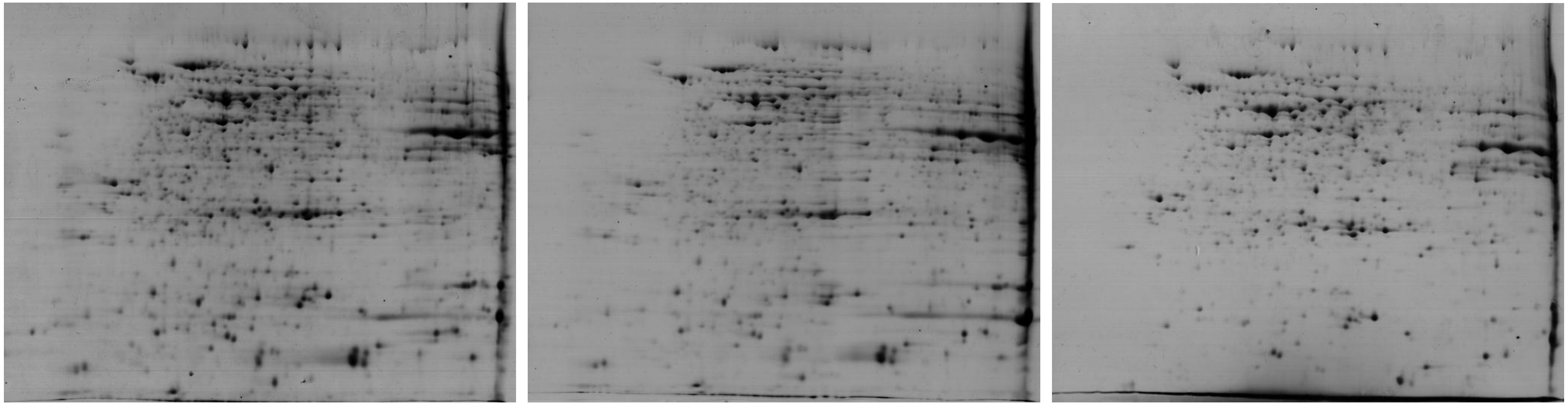

(4): 3 repeated 2D gels of PT maize seeds grown in field.

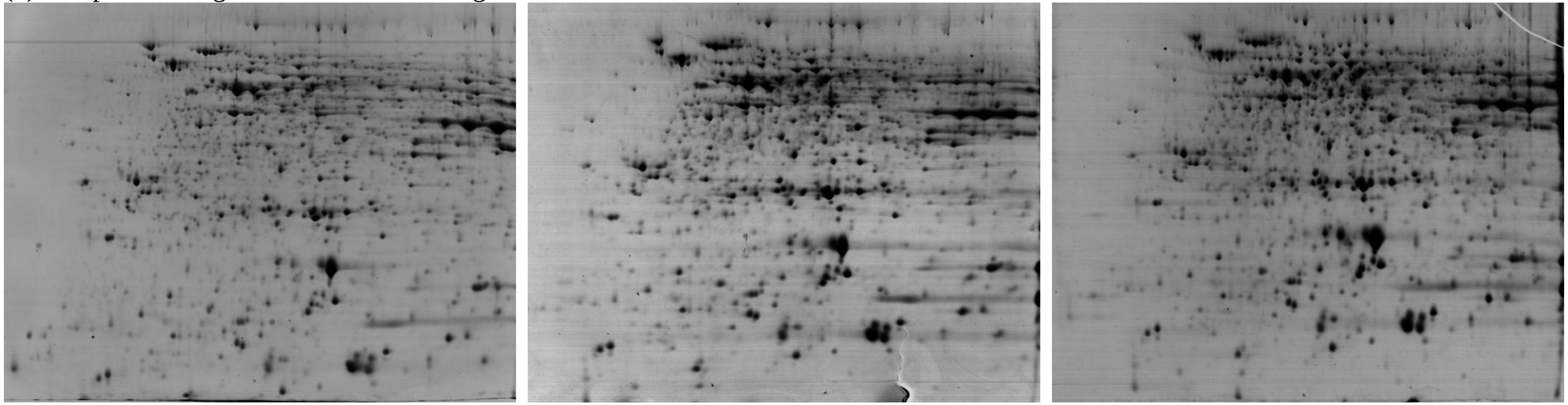

Supplementary Figure S2: Supplemental spectra and MALDI TOF/TOF MS/MS identification of the differentially expressed proteins.

Annotated spectra for Table 1: 30 proteins identified by PFF.

Spot numbers of the 30 proteins correspond to the proteins that listed in Table 1.

CID: collision induced dissociation

MALDI TOF: matrix assisted laser desorption/ionization time of flight

MS: mass spectrometry

PFF: peptide fragment fingerprinting

Spot No.: **1**

NCBI accession No.: **gi| 195630027**

Plant species: **Zea mays**

Protein name： **ruBisCO large subunit-binding protein subunit beta**

Peptide sequences: **R.DLITILEDAIR.S; K.NQIEATEQEYER.E； K.AAVEEGIVVGGGCTLLR.L； R.SGYPILIVAEDIEQEALATLVVNR.L**

PFF Mascot score: **[398]** Sequence coverage %: **[10]**

Matched peptides No.: **[4]** p value: **1.1e-09**

Calculated Mr: **64688** Calculated *p*I: **5.81**

PFF Searched Score:

Ions score is -10*Log(P), where P is the probability that the observed match is a random event.
Individual ions scores > 50 indicate identity or extensive homology (p<0.05).
Protein scores are derived from ions scores as a non-probabilistic basis for ranking protein hits.

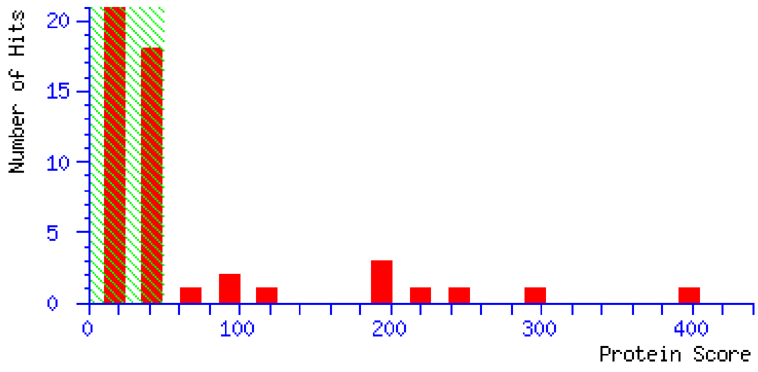

Matched peptide sequences shown in Bold Red

```
  1 MASAFGTAST CGIKHAPAGV ALAAPMKHLS LVSPSLVSLP RKMRPRRKYN
 51 FRVNAAKELH FNKDGSAIRK MQNGVNKLAD LVGVTLGPKG RNVVLESKYG
101 SPKIVNDGVT VAKEVELEDP VENIGAKLVR QAAAKTNDLA GDGTTTSVVL
151 AQGMITEGVK VVAAGANPVQ ITRGIEKTAK ALVSELQKMS KEVEDSELAD
201 VAAVSAGNNY EIGNMITEAM KKVGRQGVVT LEEGKSAENN LYSVEGMQFD
251 RGYISPYFVT DSEKMTVEYE NCKLLLVDKK INNARDLITI LEDAIRSGYP
301 ILIVAEDIEQ EALATLVVNR LRGALKIAAI KAPGFGERKS QYLDDIATLT
351 GGTVIREEVG LSLDKADNAV LGTAAKVVVT KDSTTIVGDG TTQEEVNKRV
401 TQIKNQIEAT EQEYEREKLN ERIAKLSGGV AVIQVGAQTE TELKEKKLRV
451 EDALNATKAA VEEGIVVGGG CTLLRLASKF DAIIETLEND EQKVGAEIVR
501 KSLSYPLKLI AKNAGVNGSV VTEKVLANEN FRYGYNAATG EYEDLMAAGI
551 IDPTKVVRCC LEHAASVAKT FITSDAVVVD AKVAEQAPSS PANPMAMGGG
601 SGFGF
```

Spot No.: **2**

NCBI accession No: **gi|189027076**

Plant species: **Zea mays**

Protein name: **glucose-1-phosphate adenylyltransferase large subunit 1**

Peptide sequences: **K.TPFFTAPR.C; R.ATPAVPVGGCYR.L; K.YAFISDGCLLR.E; R.SCEGDGIDRLEK.L; K.YTQLHDFGSEILPR.A; K.GIQEADHPEEGYYIR.S; K.HVEDDADITISCAPVDESR.A; R.VAATTQCILTSDACPETLHSQTQSSR.K**

PFF Mascot score: **[231]** Sequence coverage %: **[22]**

Matched peptides No.: **[8]** p value: **1.6e-04**

Calculated Mr: **57890** Calculated *p*I: **6.16**

PFF Searched Score:

Ions score is -10*Log(P), where P is the probability that the observed match is a random event.
Individual ions scores > 48 indicate identity or extensive homology (p<0.05).
Protein scores are derived from ions scores as a non-probabilistic basis for ranking protein hits.

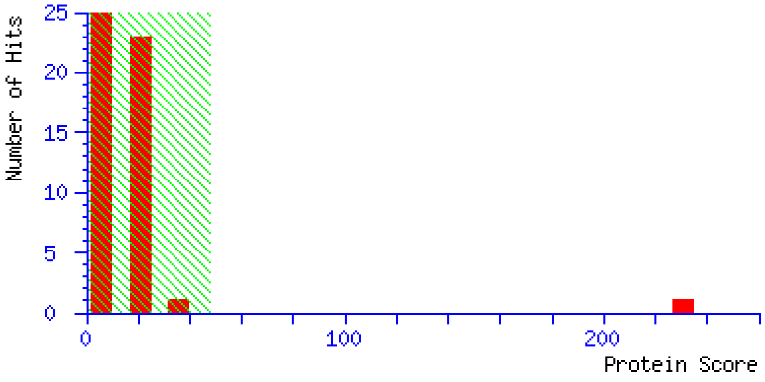

Matched peptide sequences shown in Bold Red

1	MQFALALDTN	SGPHQIR**SCE**	**GDGIDRLEK**L	SIGGRKQEKA	LRNRCFGGR**V**
51	**AATTQCILTS**	**DACPETLHSQ**	**TQSSR**KNYAD	ANRVSAIILG	GGTGSQLFPL
101	TSTR**ATPAVP**	**VGGCYR**LIDI	PMSNCFNSGI	NKIFVMSQFN	STSLNRHIHR
151	TYLEGGINFA	DGSVQVLAAT	QMPEEPAGWF	QGTADSIRKF	IWVLEDYYSH
201	KSIDNIVILS	GDQLYRMNYM	ELVQK**HVEDD**	**ADITISCAPV**	**DESR**ASKNGL
251	VKIDHTGRVL	QFFEKPKGAD	LNSMRVETNF	LSYAIDDAQK	YPYLASMGIY
301	VFKKDALLDL	LKSK**YTQLHD**	**FGSEILPR**AV	LDHSVQACIF	TGYWEDVGTI
351	KSFFDANLAL	TEQPSKFDFY	DPK**TPFFTAP**	**R**CLPPTQLDK	CKMK**YAFISD**
401	**GCLLR**ECNIE	HSVIGVCSRV	SSGCELKDSV	MMGADTYETE	EEASKLLLAG
451	KVPVGIGRNT	KIRNCIIDMN	ARIGKNVVIT	NSK**GIQEADH**	**PEEGYYIR**SG
501	IVVILKNATI	NDGSVI			

Spot No.: **3**

NCBI accession No: **gi|226503399**

Plant species: **Zea mays**

Protein name: **Elongation factor 2**

Peptide sequences: **K.DLQEDFMGGAEIIVSPPVVSFR.E; R.PLEEGLAEAIDDGR.I; R.GGGQVIPTAR.R; K.AFLPVIESFGFSSQLR.A**

PFF Mascot score: **[254]** Sequence coverage %: **[7]**

Matched peptides No.: **[4]** p value: **4.5e-07**

Calculated Mr: **94888** Calculated *p*I: **6.0**

PFF Searched Score:

Ions score is -10*Log(P), where P is the probability that the observed match is a random event.
Individual ions scores > 50 indicate identity or extensive homology (p<0.05).
Protein scores are derived from ions scores as a non-probabilistic basis for ranking protein hits.

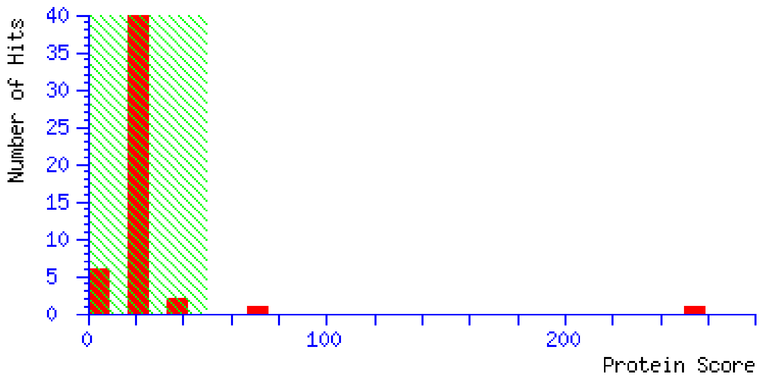

Matched peptide sequences shown in Bold Red

```
  1 MVKFTAEELR AIMDKKNNIR NMSVIAHVDH GKSTLTDSLV AAAGIIAQEV
 51 AGDVRMTDTR ADEAERGITI KSTGISLYYE MTDESLKNYK GERDGNQYLI
101 NLIDSPGHVD FSSEVTAALR ITDGALVVVD CIEGVCVQTE TVLRQALGER
151 IRPVLTVNKM DRCFLELQVE GEEAYQTFSR VIENANVIMA TYEDKLLGDV
201 QVYPEKGTVA FSAGLHGWAF TLTNFAKMYA SKFGVDETKM MERLWGENFF
251 DPATKKWTTK NTGSPTCKRG FVQFCYEPIK QIIKTCMNDQ KEKLWPMLQK
301 LNVTMKADEK ELIGKALMKR VMQTWLPAST ALLEMMIFHL PSPAKAQKYR
351 VENLYEGPLD DVYATAIRNC DPEGPLMLYV SKMIPASDKG RFFAFGRVFS
401 GKVATGMKVR IMGPNYVPGQ KKDLYVKSVQ RTVIWMGKKQ ESVEDVPCGN
451 TVAMVGLDQF ITKNATLTNE KETDACPIRA MKFSVSPVVR VAVQCKVASD
501 LPKLVEGLKR LAKSDPMVLC TMEESGEHII AGAGELHLEI CLKDLQEDFM
551 GGAEIIVSPP VVSFRETVLE KSCRTVMSKS PNKHNRLYME ARPLEEGLAE
601 AIDDGRIGPR DDPKVRSQIL SQEFGWDKDL AKKIWCFGPE TTGPNMVVDM
651 CKGVQYLNEI KDSVVAGFQW ASKEGALAEE NMRGICFEVC DVVLHADAIH
701 RGGGQVIPTA RRVIYASQLT AKPRLLEPVY LVEIQAPENA LGGIYGVLNQ
751 KRGHVFEEMQ RPGTPLYNIK AFLPVIESFG FSSQLRAATS GQAFPQCVFD
801 HWDMMGSDPL EAGSQAAQLV LDIRKRKGLK EQMTPLSEFE DKL
```

Spot No.: **4**

NCBI accession No: **gi|413949327**

Plant species: **Zea mays**

Protein name: **pyruvate, phosphate dikinase 2**

Peptide sequences: **R.SDFEGIFR.A； R.TEHMFFASDER.I； K.LYGEFLVNAQGEDVVAGIR.T； R.LLDPPLHEFLPEGNVEEIVR.E**

PFF Mascot score: **[84]**   Sequence coverage %: **[6]**

Matched peptides No.: **[4]**   p value: **1.7**
Calculated Mr: **103777**   Calculated *p*I: **6.04**

PFF Searched Score:

Ions score is -10*Log(P), where P is the probability that the observed match is a random event
Individual ions scores > 48 indicate identity or extensive homology (p<0.05).
Protein scores are derived from ions scores as a non-probabilistic basis for ranking protein hits.

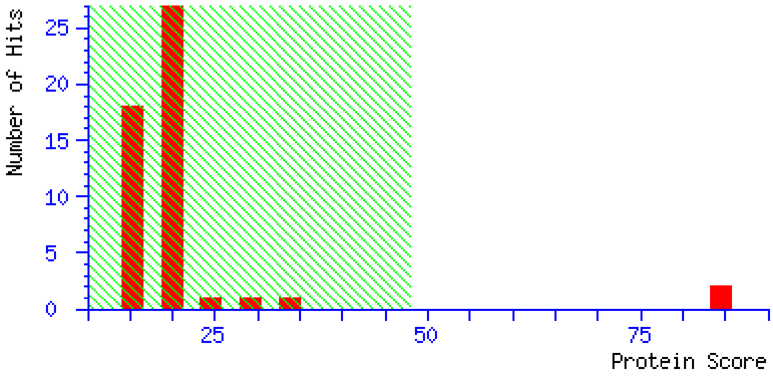

Matched peptide sequences shown in Bold Red

1	MAASVSTSRV	ICLQKPGPEG	SSRGAREVAP	FTRQSVAAPR	RPHGANASVI
51	IGSDSGAGRG	QHSPPLRAVV	DAGPVQTTKK	RVFHFGKGKS	EGNKNMKELL
101	GGKGANLAEM	ASIGLSVPPG	FTVSTEACQQ	YQEAGRALPP	GLWAEVLDGL
151	RWVEEYMGAA	LGDPRRPLLL	SVRSGAAVSM	PGMMDTVLNL	GLNDQVAAGL
201	AAKSGDRFAY	DSFRRFLDMF	GNVVMDIPHA	LFEEKLEAMK	KAKGLKNDTD
251	LTATDLKELV	SQYKNVYVEA	KGEPFPSDPK	RQLELAVLAV	FDSWESPRAK
301	KYRSINQITG	LRGTAVNVQC	MVFGNMGNTS	GTGVLFTRNP	NTGEKKLYGE
351	FLVNAQGEDV	VAGIRTPEDL	DAMKDVMPQA	YKELVENCRI	LESHYKEMQD
401	IEFTVQESRL	WMLQCRTGKR	TGKSAVKIAV	DMVNEGLVER	RAAIKMVEPG
451	HLDQLLHPQF	ENPSAYKDQV	IATGLPASPG	AAVGQVVFTA	EDAETWHSQG
501	KSVILVRAET	SPEDVGGMHA	AAGILTERGG	MTSHAAVVAR	GWGKCCVSGC
551	SGIRVNDAEK	VVKIGGNVLR	EGEWLSLNGS	TGEVILGKQP	LSPPALSGDL
601	GTFMSWVDDV	RKLKVLANAD	TPEDALAARN	NGAEGIGLCR	TEHMFFASDE
651	RIKAVRQMIM	APTVELRQQA	LDRLLPYQRS	DFEGIFRAMD	GLSVTIRLLD
701	PPLHEFLPEG	NVEEIVRELC	SETGANQEDA	LARIEKLSEV	NPMLGFRGCR
751	LGISYPELTE	MQARAIFEAA	IAMTNQGVQV	FPEIMVPLVG	TPQELGHQVA
801	LIRQVANKVF	TSMGKTIGYK	IGTMIEIPRA	ALVADEIAEQ	AEFFSFGTND
851	LTQMTFGYSR	DDVGKFIPIY	LAQGILQHDP	FEVLDQRGVG	ELVKLATERG
901	RKARPNLKVG	ICGEHGGEPS	SVAFFAKTGL	DYVSCSPFRV	PIARLAAAQV
951	LV				

Spot No.: **5**

NCBI accession No: **gi|413933276**

Plant species: **Zea mays**

Protein name: **phosphoglucomutase, cytoplasmic 1**

Peptide sequences: **K.SSSNVEPPEFGAAADGDADR.N ; R.YLFGDGSR.L ; R.VYIEQYER.D**

PFF Mascot score: **[85]** Sequence coverage %: **[5]**

Matched peptides No.: **[3]** p value: **0.026**

Calculated Mr: **70977** Calculated *p*I: **7.29**

PFF Searched Score:

Ions score is -10*Log(P), where P is the probability that the observed match is a random event.
Individual ions scores > 48 indicate identity or extensive homology ($p<0.05$).
Protein scores are derived from ions scores as a non-probabilistic basis for ranking protein hits.

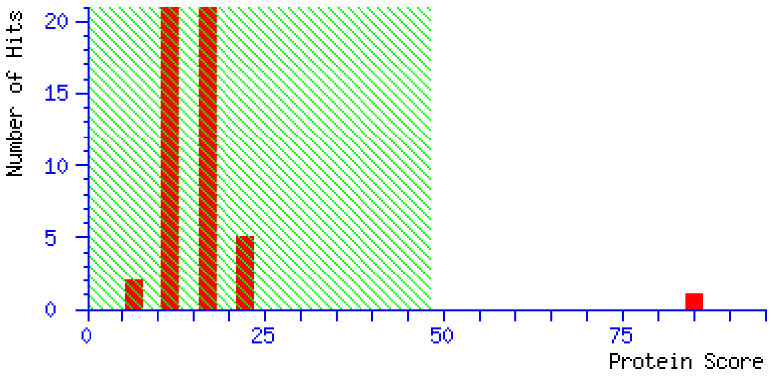

Matched peptide sequences shown in Bold Red

1 MIQHRPLVLS LPSPAIIPPR PRRHRHHTTT PSLPSHSRSL LHHRFLRVTH
51 LVVASRGDQR RTLRGATMGL FTVTKKATTP FDGQKPGTSG LRKKVTVFQQ
101 PHYLQNFVQS TFNALPVDQV RGATIVVSGD GRYFSKDAVQ IITKMAAANG
151 VRRVWVGQNS LMSTPAVSAV IRERVGADGS KATGAFILTA SHNPGGPKED
201 FGIKYNMGNG GPAPESVTDK IFSNTTTISE YLISEDLPDV DISVVGVTSF
251 SGPEGPFDVD VFDSSVDYIK LMKTIFDFEA IKKLLTSPKF TFCYDALHGV
301 AGAYAKHIFV EELGADESSL LNCVPKEDFG GGHPDPNLTY AKELVERMGL
351 GK**SSSNVEPP EFGAAADGDA DR**NMILGKRF FVTPSDSVAI IAANAVQSIP
401 YFASGLKGVA RSMPTSAALD VVAKNLNLKF FEVPTGWKFF GNLMDAGMCS
451 ICGEESFGTG SDHIREKDGI WAVLAWLSII AFKNKDNLGG DKLVTVEDIV
501 RQHWATYGRH YYTRYDYENV DAGAAKELMA NLVSMQSSLS DVNKLVKEIR
551 SDVSEVVAAD EFEYKDPVDG SVSKHQGIR**Y LFGDGSR**LVF RLSGTGSVGA
601 TIR**VYIEQYE R**DSSKTGRDS QDALAPLVDV ALKLSKMQEY TGRSAPTVIT

Spot No.: **6**

NCBI accession No: **gi|212275400**

Plant species: ***Zea mays***
Protein name: **uncharacterized protein LOC100191408**

Peptide sequences: **K.AVITVPAYFNDAQR.Q; K.SQVFSTAADNQTQVGIR.V**

PFF Mascot score: **[63]** Sequence coverage %: **[4]**

Matched peptides No.: **[2]** p value: **0.9**
Calculated Mr:**72903** Calculated *p*I: **5.54**

PFF Searched Score:

Ions score is -10*Log(P), where P is the probability that the observed match is a random event.
Individual ions scores > 48 indicate identity or extensive homology (p<0.05).
Protein scores are derived from ions scores as a non-probabilistic basis for ranking protein hits.

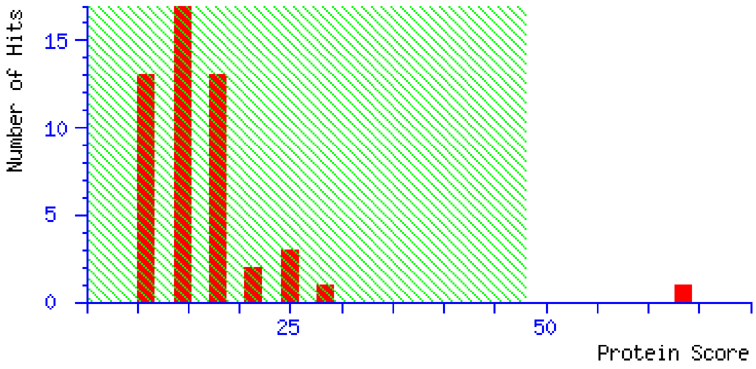

Matched peptide sequences shown in Bold Red

1 MAASLLLRAV RRRELASPLG SLGANLQSTC AANICSKWGN FARPFSAKAA
51 GNEVIGIDLG TTNSCVAVME GKNPKVIENA EGARTTPSVV AFTQKGERLV
101 GTPAKRQAVT NPQNTFFGTK RMIGRRFDDP QTQKEMKMVP YKIVKAPNGD
151 AWVETTDGKQ YSPSQVGAFV LTKMKETAES YLGKSVSK**AV** **ITVPAYFNDA**
201 **QR**QATKDAGR IAGLDVERII NEPTAAALSY GMNNKEGLIA VFDLGGGTFD
251 ISILEISNGV FEVKATNGDT FLGGEDFDNT LLEFLVSDFK KTEGIDLSKD
301 RLALQRLREA AEKAKVELSS TSQTEINLPF ITADASGAKH LNITLTRSKF
351 ESLVHNLIER TRDPCKNCLK DAGISTKEVD EVLLVGGMTR VPKVQEVVSE
401 IFGKSPSKGV NPDEAVAMGA AIQGGILRGD VKELLLLDVT PLSLGIETLG
451 GIFTRLINRN TTIPTKK**SQV** **FSTAADNQTQ** **VGIR**VLQGER EMAADNKLLG
501 EFDLVGIPPA PRGLPQIEVA FDIDANGIVT VAAKDKATGK EQNITIRSSG
551 GLSEADIQKM VQEAELHAQK DQERKALIDI RNTADTTIYS IEKSLGEYRD
601 KIPAEVASEI EAAIADLRQE MASDDIEKIK AKLEAANKAV SKIGQHMSGG
651 GSGDSQSGSG PQGGGDQAPE AEYEEVKK

Spot No.: **7**

NCBI accession No: **gi|226509912**

Plant species: **Zea mays**

Protein name: **ubiquitin carboxyl-terminal hydrolase 6**

Peptide sequences: **R.YLTVQFVR.F; K.WIEFDDDNPNIR.K**

PFF Mascot score: **[99]** Sequence coverage %: **[4]**

Matched peptides No.: **[2]** p value: **0.0044**

Calculated Mr:**53978** Calculated pI: **5.73**

PFF Searched Score:

Ions score is -10*Log(P), where P is the probability that the observed match is a random event.
Individual ions scores > 50 indicate identity or extensive homology (p<0.05).
Protein scores are derived from ions scores as a non-probabilistic basis for ranking protein hits.

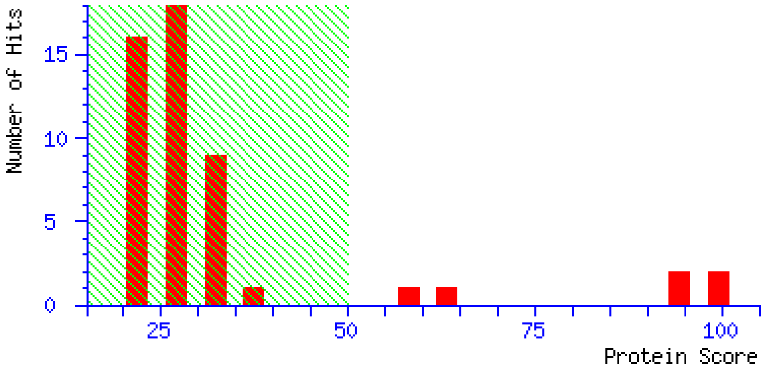

Matched peptide sequences shown in Bold Red

```
  1 MPTVSVKWQK ELFPGIEIDT SQPPIVFKTQ LYTLTGVPPE RQKIMVKGGI
 51 LKDDADWSTL GVKDGQKLMM IGTADEIVKA PEKGPVFVED LPEEEQVIAL
101 GHSAGLYNLG NTCYMNSTLQ CLHSVPELKS ALLSYSDTVR GNGIDQASHN
151 LTVATRNTFG DLDQSVRPVA PLQFLQTLRK KYPQFAQQHN NVYMQQDAEE
201 CWTQLVYTLS QTLTSDSSES AALPMKQLFG IDLVSRVHCA ESGEESMETE
251 SVYSLKCHIS QDVNHLHEGI KHGLKTELEK ASPTLGRTAV YTRESRINEL
301 PRYLTVQFVR FFWKRESNQK AKILRKVDYP LELDVYEFCS DELKQKLQAP
351 RQMLRDAENA KFGLKAQGKA SGFKENEGSS SSAGESSSMD IDKAESSLPK
401 KQLTGVYDLV AVLTHKGRSA DSGHYVGWVK QDDGKWIEFD DDNPNIRKEE
451 DILKLSGGGD WHMAYICLYK ARMAELKS
```

Spot No.: **8**

NCBI accession No: **gi|189027076**

Plant species: **Zea mays**

Protein name： **glucose-1-phosphate adenylyltransferase large subunit 1**

Peptide sequences: **R.VAATTQCILTSDACPETLHSQTQSSR.K； R.VSAIILGGGTGSQLFPLTSTR.A； R.ATPAVPVGGCYR.L ； K.SIDNIVILSGDQLYR.M； K.HVEDDADITISCAPVDESR.A； K.TPFFTAPR.C； K.GIQEADHPEEGYYIR.S**

PFF Mascot score: **[424]** Sequence coverage %: **[22]**

Matched peptides No.: **[7]** p value: **7.2e-08**

Calculated Mr: **57890** Calculated *p*I: **6.16**

PFF Searched Score:

Ions score is -10*Log(P), where P is the probability that the observed match is a random event.
Individual ions scores > 48 indicate identity or extensive homology (p<0.05).
Protein scores are derived from ions scores as a non-probabilistic basis for ranking protein hits.

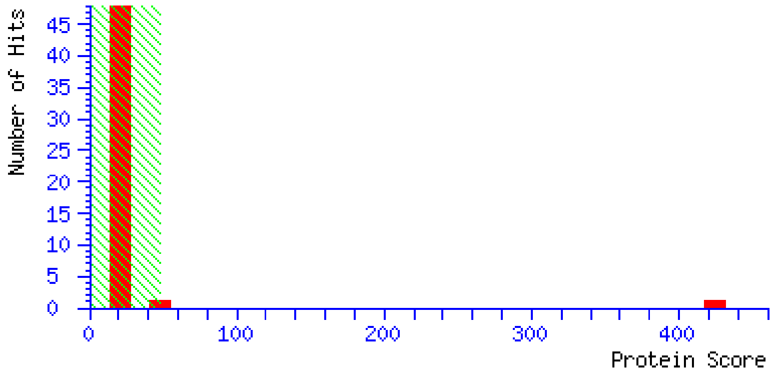

Matched peptide sequences shown in Bold Red

1 MQFALALDTN SGPHQIRSCE GDGIDRLEKL SIGGRKQEKA LRNRCFGGRV
51 AATTQCILTS DACPETLHSQ TQSSRKNYAD ANRVSAIILG GGTGSQLFPL
101 TSTRATPAVP VGGCYRLIDI PMSNCFNSGI NKIFVMSQFN STSLNRHIHR
151 TYLEGGINFA DGSVQVLAAT QMPEEPAGWF QGTADSIRKF IWVLEDYYSH
201 KSIDNIVILS GDQLYRMNYM ELVQKHVEDD ADITISCAPV DESRASKNGL
251 VKIDHTGRVL QFFEKPKGAD LNSMRVETNF LSYAIDDAQK YPYLASMGIY
301 VFKKDALLDL LKSKYTQLHD FGSEILPRAV LDHSVQACIF TGYWEDVGTI
351 KSFFDANLAL TEQPSKFDFY DPKTPFFTAP RCLPPTQLDK CKMKYAFISD
401 GCLLRECNIE HSVIGVCSRV SSGCELKDSV MMGADTYETE EEASKLLLAG
451 KVPVGIGRNT KIRNCIIDMN ARIGKNVVIT NSKGIQEADH PEEGYYIRSG
501 IVVILKNATI NDGSVI

Spot No.: **9**

NCBI accession No: **gi|413956739**

Plant species: **Zea mays**

Protein name: **myo-inositol phosphate synthase**

Peptide sequences: **-.MFIESFR.V; R.YGPTEIESEYR.Y; K.DKVQQANYYGSLTQASTIR.V; K.VVVLWTANTER.Y**

PFF Mascot score: **[204]** Sequence coverage %: **[10]**

Matched peptides No.: **[4]** p value: **4e-08**

Calculated Mr: **50215** Calculated pI: **5.46**

PFF Searched Score:
Ions score is -10*Log(P), where P is the probability that the observed match is a random event.
Individual ions scores > 50 indicate identity or extensive homology (p<0.05).
Protein scores are derived from ions scores as a non-probabilistic basis for ranking protein hits.

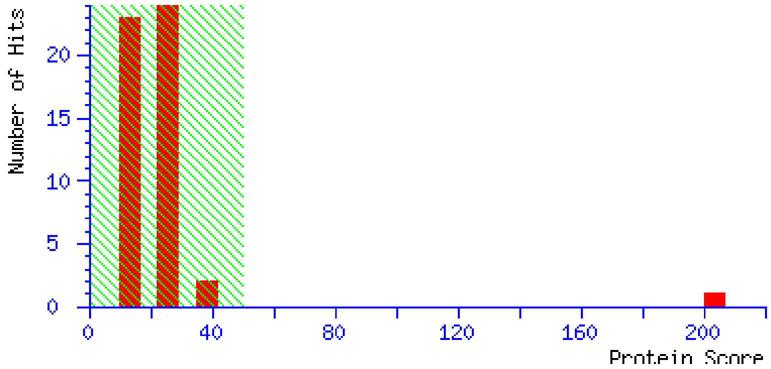

Matched peptide sequences shown in Bold Red

```
  1 MFIESFRVES PHVRYGPTEI ESEYRYDTTE LVHEAKDGAS RWVVRPKSVK
 51 YNFRTSTAVP KLGVMLVGWG GNNGSTLTAG VIANREGISW ATKDKVQQAN
101 YYGSLTQAST IRVGSYNGEE IYAPFKSLLP MVNPDDLVFG GWDISSMNLA
151 DAMTRAKVLD IDLQKQLRPY MESMVPLPGV YDPDFIAANQ GSRANNVIKG
201 TKKEQVEQII KDIREFKEKN KVDKVVVLWT ANTERYSNVC AGLNDTMENL
251 LASVDKNEAE ISPSTLYAIA CVTEGVPFIN GSPQNTFVPG LIDLAIKNNC
301 LIGGDDFKSG QTKMKSVLVD FLVGAGIKPT SIVSYNHLGN NDGMNLSAPQ
351 TFRSKEISKS NVVDDMVSSN AILYGPGEHP DHVVVIKYVP YVGDSKRAMD
401 EYTSEIFMGG KSTIVLHNTC EDSLLAAPII LDLVLLAELS TRIQLKPEGT
451 VRAHE
```

Spot No.: **10**

NCBI accession No: **gi|162460991**

Plant species: **Zea mays**

Protein name： **indole-3-acetate beta-glucosyltransferase**

Peptide sequences: **R.NVELAWGAGVR.A; R.NLDEFVQFVR.A;**

PFF Mascot score: **[90]** Sequence coverage %: **[4]**

Matched peptides No.: **[2]** p value: **0.091**

Calculated Mr: **50135** Calculated *p*I: **5.75**

PFF Searched Score:

Ions score is -10*Log(P), where P is the probability that the observed match is a random event.
Individual ions scores > 51 indicate identity or extensive homology (p<0.05).
Protein scores are derived from ions scores as a non-probabilistic basis for ranking protein hits.

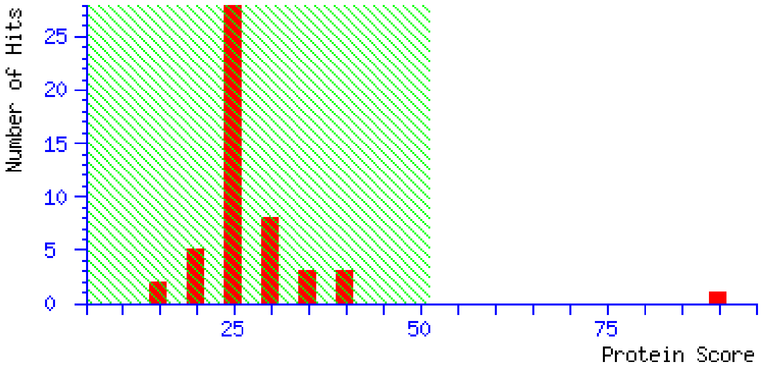

Matched peptide sequences shown in **Bold Red**

```
  1 MAPHVLVVPF PGQGHMNPMV QFAKRLASKG VATTLVTTRF IQRTADVDAH
 51 PAMVEAISDG HDEGGFASAA GVAEYLEKQA AAASASLASL VEARASSADA
101 FTCVVYDSYE DWVLPVARRM GLPAVPFSTQ SCAVSAVYYH FSQGRLAVPP
151 GAAADGSDGG AGAAALSEAF LGLPEMERSE LPSFVFDHGP YPTIAMQAIK
201 QFAHAGKDDW VLFNSFEELE TEVLAGLTKY LKARAIGPCV PLPTAGRTAG
251 ANGRITYGAN LVKPEDACTK WLDTKPDRSV AYVSFGSLAS LGNAQKEELA
301 RGLLAAGKPF LWVVRASDEH QVPRYLLAEA TATGAAMVVP WCPQLDVLAH
351 PAVGCFVTHC GWNSTLEALS FGVPMVAMAL WTDQPTNARN VELAWGAGVR
401 ARRDAGAGVF LRGEVERCVR AVMDGGEAAS AARKAAGEWR DRARAAVAPG
451 GSSDRNLDEF VQFVRAGATE K
```

Spot No.: **11**

NCBI accession No: **gi|2500522**

Plant species: **Zea mays**

Protein name: **eukaryotic initiation factor 4A (eIF4A)**

Peptide sequences: **R.GIYAYGFEKPSAIQQR.G; K.TATFCSGILQQLDYGLVECQALVLAPTR.E; R.ILASGVHVVVGTPGR.V; K.IQVGVFSATMPPEALEITR.K; K.LDTLCDLYETLAITQSVIFVNTR.R; R.GIDVQQVSLVINYDLPTQPENYLHR.I**

PFF Mascot score: **[773]** Sequence coverage %: **[30]**

Matched peptides No.: **[6]** p value: **1.5e-13**

Calculated Mr:**46849** Calculated *p*I: **5.28**

PFF Searched Score:

Ions score is -10*Log(P), where P is the probability that the observed match is a random event.
Individual ions scores > 50 indicate identity or extensive homology (p<0.05).
Protein scores are derived from ions scores as a non-probabilistic basis for ranking protein hits.

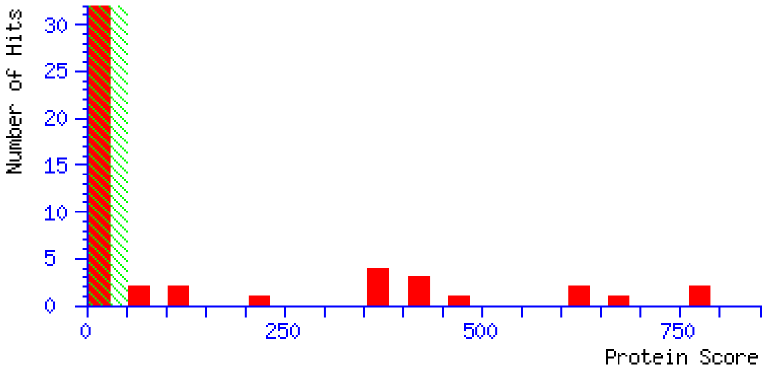

Matched peptide sequences shown in Bold Red

```
  1 MAGLAPEGSQ FDDKQYDKKM QEILTEDFFT SYDDVCESFD SMGLQENLLR
 51 GIYAYGFEKP SAIQQRGIVP FCKGLDVIQQ AQSGTGKTAT FCSGILQQLD
101 YGLVECQALV LAPTRELAQQ IEKVMRALGD YLGVKVHACV GGTSVREDQR
151 ILASGVHVVV GTPGRVFDML RRQSLRPDNI KMFVLDEADE MLSRGFKDQI
201 YDIFQLLPSK IQVGVFSATM PPEALEITRK FMNKPVRILV KRDELTLEGI
251 KQFYVNIDKE DWKLDTLCDL YETLAITQSV IFVNTRRKVD WLTDKMRSRD
301 HTVSATHGDM DQNTRDIIMR EFRSGSSRVL ITTDLLARGI DVQQVSLVIN
351 YDLPTQPENY LHRIGRSGRF GRKGVAINFV TRDDERIVFD VQRFYNVTVE
401 ELPANVADLL
```

Spot No.: **12**

NCBI accession No: **gi|226529884**

Plant species: **Zea mays**

Protein name: **10-deacetylbaccatin III 10-O-acetyltransferase**

Peptide sequences: K.ALVPYYPVAGR.I; R.GLPAPSVAPVWDR.D; K.EELLPRPPPEEK.L

PFF Mascot score: **[171]** Sequence coverage %: **[8]**

Matched peptides No.: **[3]** p value: **1.9e-03**

Calculated Mr: **45911** Calculated *p*I: **5.03**

PFF Searched Score:

Ions score is -10*Log(P), where P is the probability that the observed match is a random event.
Individual ions scores > 51 indicate identity or extensive homology (p<0.05).
Protein scores are derived from ions scores as a non-probabilistic basis for ranking protein hits.

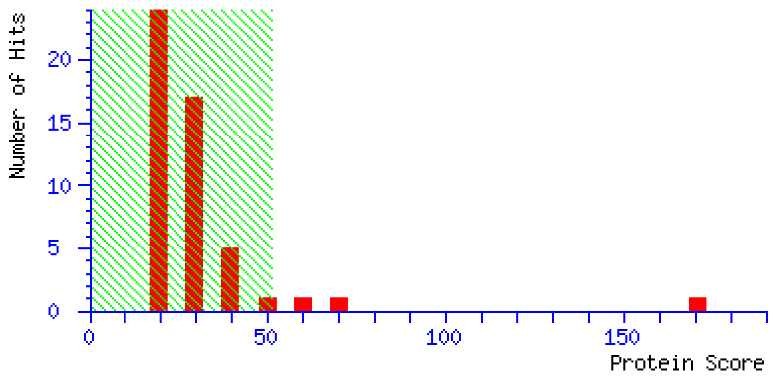

Matched peptide sequences shown in **Bold Red**

```
  1 MSTVAAAPTV VKSAPELVAP VGPTPGGTLP LSSIDKTAAV RVSVDFIQVF
 51 PPAVGAGGDK DAAVAAMRDG FAKALVPYYP VAGRIADASP GEPVVECTGQ
101 GVWFVEAAAS CALADVNYLE RPLLIPKEEL LPRPPPEEKL EDLVLMAQVT
151 KFACGGFAVG ICFSHLVFDG QGAAQFLKAA GEMARGLPAP SVAPVWDRDA
201 IPDPPKLPRG PPPSFTAFSF VTQVVEISPE SIARIKDDFK DATGQTCSTF
251 DAVTAVVFKC RALAMALPDD AEVRLGFAAS TRHLLHGVLP SVDGYYGNCV
301 YPVGITRTSK AIREASLPEV VGVMREAKEA LTTRFTDWMR GGAKDDHYNV
351 PLDYGTVTVS DWSRVGFNEV DYGFGEPGYV FTLNDDVNIV ASVIYLKPPA
401 PKRGIRLMLR CVEEPHAAAF AEELAKFA
```

Spot No.: **13**

NCBI accession No: **gi|413950795**

Plant species: **Zea mays**

Protein name: **Isocitrate dehydrogenase [NADP]**

Peptide sequences: **R.HAFGDQYR.A; K.YYDLGILHR.E; K.DLAILVHGSSSVTR.S; K.SKFEAAGIWYEHR.L; R.SHYLNTEEFIDAVATELR.S**

PFF Mascot score: **[270]**   Sequence coverage %: **[15]**

Matched peptides No.: **[5]**   p value: **3.1e-09**

Calculated Mr:**46510**   Calculated pI: **6.11**

PFF Searched Score:

Ions score is -10*Log(P), where P is the probability that the observed match is a random event.
Individual ions scores > 51 indicate identity or extensive homology (p<0.05).
Protein scores are derived from ions scores as a non-probabilistic basis for ranking protein hits.

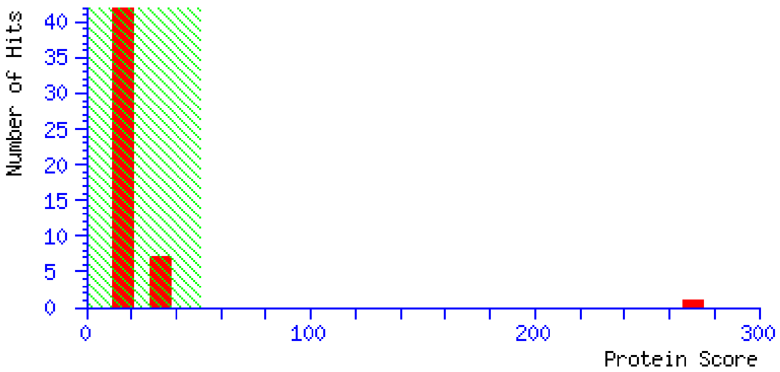

Matched peptide sequences shown in Bold Red

```
  1 MAFEKIKVSN PIVEMDGDEM TRVFWQSIKD KLILPFLDLD IKYYDLGILH
 51 REATDDKVTV EAAEATLKYN VAIKCATITP DETRVKEFNL KHMWKSPNGT
101 IRNIINGTVF REPIICKNVP RLVPGWTKPI CIGRHAFGDQ YRATDAVLKG
151 PGKLKLVFEG KEEQIDLEVF NFTGAGGVAL SMYNTDESIR AFAEASMTTA
201 YEKKWPLYLS TKNTILKKYD GRFKDIFQEV YEADWKSKFE AAGIWYEHRL
251 IDDMVAYALK SEGGYVWACK NYDGDVQSDF LAQGFGSLGL MTSVLVCPDG
301 KTIEAEAAHG TVTRHFRVHQ KGGETSTNSI ASIFAWTRGL AHRAKLDDNA
351 RLLDFALKLE AACVETVESG KMTKDLAILV HGSSSVTRSH YLNTEEFIDA
401 VATELRSRLG AN
```

Spot No.: 14

NCBI accession No: **gi|195627248**

Plant species: **Zea mays**

Protein name: **sorbitol dehydrogenase**

Peptide sequences: **K.LPPVGPYDVR.V; K.AVGICGSDVHYLR.E; R.AGVGPETGVLVVGAGPIGLVSLLAAR.A; K.EPMVIGHECAGVVEEVGAGVTHLSVGDR.V**

PFF Mascot score: **[413]** Sequence coverage %: **[21]**

Matched peptides No.: **[4]** p value: **4.5e-15**

Calculated Mr:**39528** Calculated pI: **6.27**

PFF Searched Score:

Ions score is -10*Log(P), where P is the probability that the observed match is a random event.
Individual ions scores > 51 indicate identity or extensive homology (p<0.05).
Protein scores are derived from ions scores as a non-probabilistic basis for ranking protein hits.

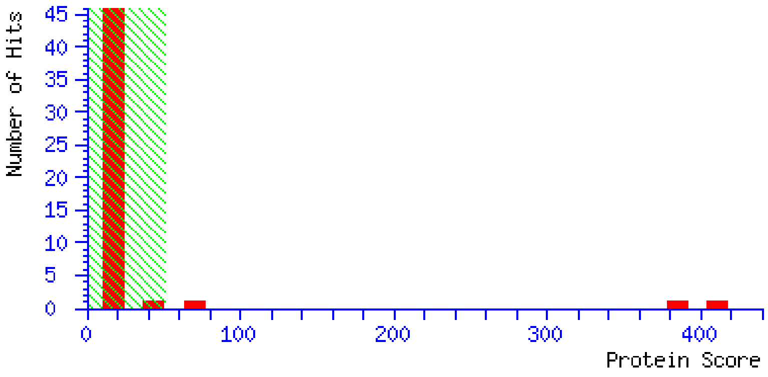

Matched peptide sequences shown in Bold Red

1 MGKGAQGSDA AAAGGEVEEN MAAWLVAKNT LKIMPFK**LPP** **VGPYDVR**VRM
51 K**AVGICGSDV** **HYLR**EMRIAH FVVK**EPMVIG** **HECAGVVEEV** **GAGVTHLSVG**
101 **DR**VALEPGVS CWRCRHCKGG RYNLCEDMKF FATPPVHGSL ANQVVHPADL
151 CFKLPDGVSL EEGAMCEPLS VGVHACRR**AG** **VGPETGVLVV** **GAGPIGLVSL**
201 **LAAR**AFGAPR VVVVDVDDHR LAVARSLGAD AAVRVSPRAE DLADEVERIR
251 AAMGSDIDVS LDCAGFSKTM STALEATRPG GKVCLVGMGH NEMTLPLTAA
301 AAREVDVVGF RYKDTWPLCI DFLRSGKVDV KPLITHRFGF SQRDVEEAFE
351 VSARGRDAIK VMFNL

Spot No.: 15

NCBI accession No: **gi|670397371**

Plant species: ***Zea mays***

Protein name: **PREDICTED: adenosine kinase 2-like**

Peptide sequences: **K.NAQAAGVTAHYYEDETAPTGTCAVCVVGGER.S; K.RPENWALVEK.A; K.LVDTNGAGDAFVGGFLSR.L**

PFF Mascot score: **[252]** Sequence coverage %: **[17]**

Matched peptides No.: **[3]** p value: **3.8e-08**

Calculated Mr: **37436** Calculated pI: **5.00**

PFF Searched Score:

Ions score is -10*Log(P), where P is the probability that the observed match is a random event.
Individual ions scores > 50 indicate identity or extensive homology (p<0.05).
Protein scores are derived from ions scores as a non-probabilistic basis for ranking protein hits.

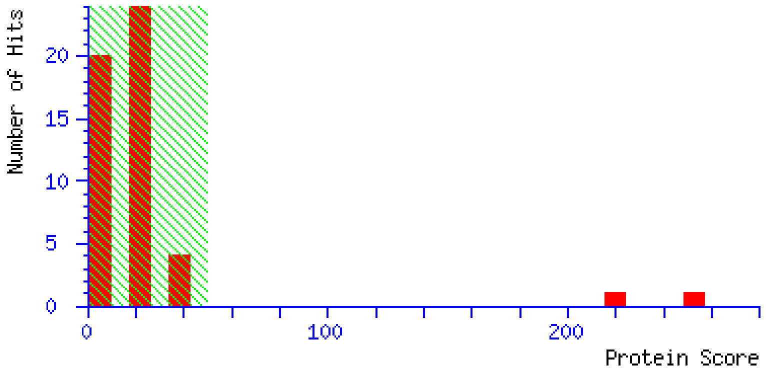

Matched peptide sequences shown in Bold Red

```
  1 MAASEGVLLG MGNPLLDISA VVDDAFLAKY DIKLNNAILA EEKHSPMYDE
 51 LASNSNVEYI AGGATQNSIR VAQWMLQTPG ATSYMGCIGK DKFGEEMKKN
101 AQAAGVTAHY YEDETAPTGT CAVCVVGGER SLIANLSAAN CYKSEHLKRP
151 ENWALVEKAK YIYIAGFFLT VSPDSIQLVA EHAAANNKVF LMNLSAPFIC
201 EFFYDAQEKV LPYADFIFGN ETEAKIFAKV RGWETENVEE IALKISQLPL
251 ASGKQKRIAV ITQGADPVVV AEDGKVKTFP VILLPKEKLV DTNGAGDAFV
301 GGFLSRLVQG KSIEDCVTAG CYAANVVIQR PGCTYPEKPD FN
```

Spot No.: **16**

NCBI accession No: **gi|195644252**

Plant species: **Zea mays**

Protein name: **aspartate-semialdehyde dehydrogenase**

Peptide sequences: **R.GLDIFVCGDQIR.K; R.FPTPLEVSDKDDVAVGR.I; K.YGPAAVASGAVVVDNSSAFR.M; R.KYGPAAVASGAVVVDNSSAFR.M; R.EYTVQDLAAPGAFDGVDIALFSAGGSVSR.K**

PFF Mascot score: **[413]** Sequence coverage %: **[21]**

Matched peptides No.: **[5]** p value: **1.7e-11**

Calculated Mr:**40833** Calculated pI: **6.62**

PFF Searched Score:

Ions score is -10*Log(P), where P is the probability that the observed match is a random event.
Individual ions scores > 50 indicate identity or extensive homology (p<0.05).
Protein scores are derived from ions scores as a non-probabilistic basis for ranking protein hits.

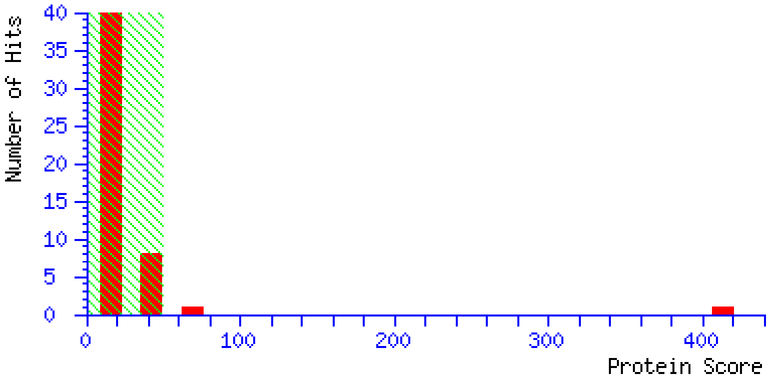

Matched peptide sequences shown in Bold Red

```
  1 MQAAAAVRHP RLFPPSPGSS AQHRRAPSTV RMALREDGPS VAIVGATGAV
 51 GQEFLRVITD RDFPYRGLRL LASERSAGKR LAFEDREYTV QDLAAPGAFD
101 GVDIALFSAG GSVSRKYGPA AVASGAVVVD NSSAFRMEPE VPLVIPEVNP
151 EAMANVRLGQ GAIVANPNCS TIICLMAATP LHRHAKVLRM VVSTYQAASG
201 AGAAAMEELK LQTQEVLEGK APTCNIFKQQ YAFNIFSHNA PVLENGYNEE
251 EMKMVKETRK IWNDKAVKVT ATCIRVPVMR AHAESVNLQF EKPLDEDTAR
301 EILRAAPGVT IIDDRASNRF PTPLEVSDKD DVAVGRIRQD LSLDDNRGLD
351 IFVCGDQIRK GAALNAVQIA EMLLK
```

Spot No.: **17**

NCBI accession No: **gi|162464283**

Plant species: ***Zea mays***

Protein name： **homocysteine S-methyltransferase 3**

Peptide sequences: **K.SVQIALEAR.E; R.FIHGLILSIR.K; K.CLLSSPHLIR.K; K.VGAVGINCTPPR.F**

PFF Mascot score: **[159]** Sequence coverage %: **[12]**

Matched peptides No.: **[4]** p value: **0.13**

Calculated Mr:**37247** Calculated pI: **5.53**

PFF Searched Score:

Ions score is -10*Log(P), where P is the probability that the observed match is a random event.
Individual ions scores > 51 indicate identity or extensive homology (p<0.05).
Protein scores are derived from ions scores as a non-probabilistic basis for ranking protein hits.

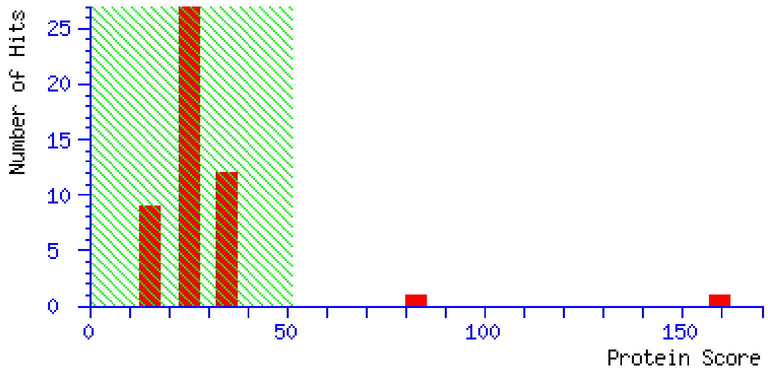

Matched peptide sequences shown in Bold Red

1 MVGTAEGGAE RAVRRWVDAA GGRLVLDGGL ATELEANGAD LNDPLWSAK**C**

51 **LLSSPHLIR**K VHMDYLEAGA NIIITASYQA TIQGFESKGF SKEQSENLLT

101 K**SVQIALEAR** EMFLKEHLEK STPIQHPILV AAALGSYGAY LADGSEYSGD

151 YGEAGTKEFL KDFHRRRLQV LAEAGPDLIA FETIPNKLEA QAYVELLEEC

201 NINIPSWLSF NSKDGVHVVS GDSLIECATI ADKCAK**VGAV** **GINCTPPRFI**

251 **HGLILSIR**KV TDKPILIYPN SGERYDGEKK EWVESTGVSD GDFVSYVNEW

301 CKDGAALIGG CCRTTPNTIR AIHRTLNQGC HKHQLPVA

Spot No.: **18**

NCBI accession No: **gi|195628698**

Plant species: **Zea mays**

Protein name: **hypothetical protein**

Peptide sequences: **R.YPPALELPIPR.L; R.SLQITLVGGSIAER.S; K.TLTAGQSPTVVDTDVGR.I; R.AADNQLFVATCAPAR.D**

PFF Mascot score: **[186]** Sequence coverage %: **[18]**

Matched peptides No.: **[4]** p value: **1.3e-06**

Calculated Mr:**34244** Calculated pI: **5.92**

PFF Searched Score:

Ions score is -10*Log(P), where P is the probability that the observed match is a random event.
Individual ions scores > 48 indicate identity or extensive homology (p<0.05).
Protein scores are derived from ions scores as a non-probabilistic basis for ranking protein hits.

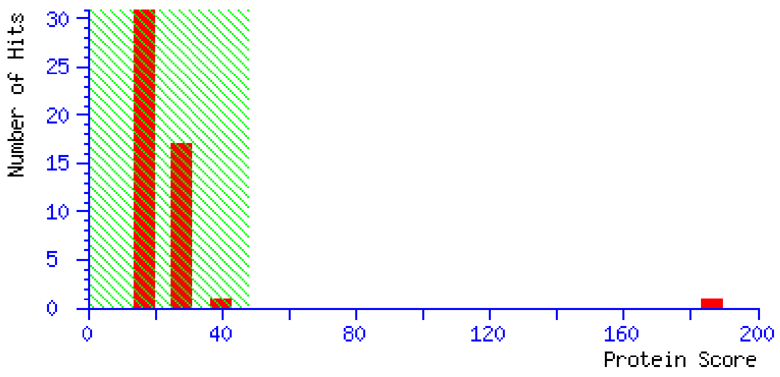

Matched peptide sequences shown in Bold Red

```
  1 MASAPNSSFR PEEARYPPAL ELPIPRLSKF KVALCQLSVT ADKSRNIAHA
 51 RAAIEKAASD GAKLVVLPEI WNGPYSNDSF PEYAEDIEAG GDAAPSFSML
101 SEVARSLQIT LVGGSIAERS GNNLYNTCCV FGSDGQLKGK HRKIHLFDID
151 IPGKITFKES KTLTAGQSPT VVDTDVGRIG IGICYDIRFQ ELAMLYAARG
201 AHLLCYPGAF NMTTGPLHWE LLQRARAADN QLFVATCAPA RDTSAGYVAW
251 GHSTLVGPFG EVIATTEHEE ATIIADIDYS LIEQRRQFLP VQHQRRGDLY
301 QLVDVQRLGS Q
```

Spot No.: **19**

NCBI accession No: **gi|806638661**

Plant species: **Zea mays**

Protein name: **cysteine synthase**

Peptide sequences: **K.LFVVVFPSFGER.Y; K.EGLLVGISSGAAAAAAVR.L**

PFF Mascot score: **[98]** Sequence coverage %: **[9]**

Matched peptides No.: **[2]** p value: **4.4e-007**

Calculated Mr:**34266** Calculated pI: **5.67**

PFF Searched Score:

Ions score is -10*Log(P), where P is the probability that the observed match is a random event.
Individual ions scores > 50 indicate identity or extensive homology (p<0.05).
Protein scores are derived from ions scores as a non-probabilistic basis for ranking protein hits.

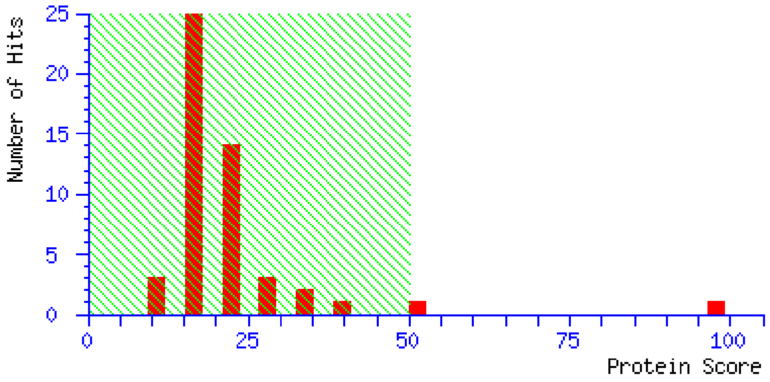

Matched peptide sequences shown in Bold Red

1 MGEASPSIAK DVTELIGNTP LVYLNKVTDG CVGRVAAKLE SMEPCSSVKD
51 RIGYSMITDA EEKGLITPGV SVLIEPTSGN TGIGLAFMAA AKGYKLTLTM
101 PASMSMERRI ILKAFGAELV LTDPLLGMKG AVKKAEEIQA KTPNSYILQQ
151 FENPANPKIH YETTGPEIWK ATAGKIDGLV SGIGTGGTIT GTGRYLREQN
201 PNVKLYGVEP VESAVLNGGK PGPHKIQGIG AGFIPGVLDV DLLDETLQVS
251 SDEAIETAKA LALK**EGLLVG** **ISSGAAAAAA** **VR**LAKRPENA GK**LFVVVFPS**
301 **FGER**YLSSVL FQSIKKEAES MVVEP

Spot No.: **20**

NCBI accession No: **gi|162460029**

Plant species: ***Zea mays***

Protein name: **glutathione transferase41**

Peptide sequences: **R.LVDADAFPR.F; R.DFEALDEVR.E; R.QVHENLALIESELR.D; K.LGPAVGAVFASTGEGQEAAVR.Q**

PFF Mascot score: **[153]** Sequence coverage %: **[19]**

Matched peptides No.: **[4]** p value: **0.024**

Calculated Mr: **29093** Calculated pI: **4.85**

PFF Searched Score:

Ions score is -10*Log(P), where P is the probability that the observed match is a random event.
Individual ions scores > 49 indicate identity or extensive homology (p<0.05).
Protein scores are derived from ions scores as a non-probabilistic basis for ranking protein hits.

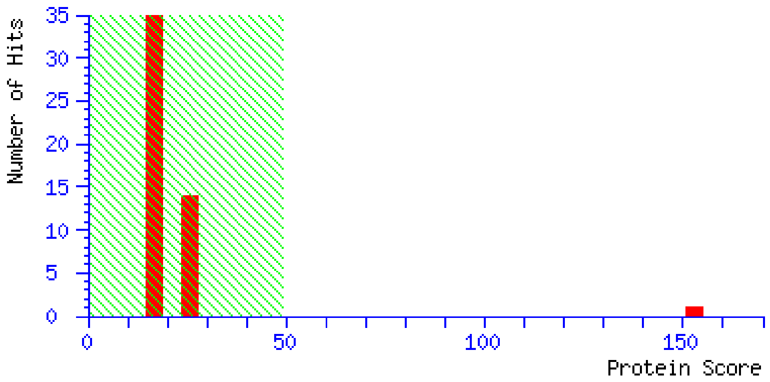

Matched peptide sequences shown in Bold Red

```
  1  MEKTSESAIP PAASPLMLFG SWASSYTHRV QLALRLKGLE FDYVEEDLGN
 51  KSDELLRHNP VHKKVPVLVH GGRALPESVI ILQYLDDAWP ETRPLLPADA
101  FDRALARFWC HFADDKLGPA VGAVFASTGE GQEAAVRQVH ENLALIESEL
151  RDGAFRGRRF FGGDEVGLLD VVLGCGSYWL AVFEEVTGVR LVDADAFPRF
201  HAWLRDFEAL DEVRETIPAV DRLLEYARGL RHMLLGLAGA GAGAGADAPS
251  TDAPAAPPSS ADIAVDI
```

Spot No.: **21**

NCBI accession No: **gi|226495167**

Plant species: ***Zea mays***

Protein name： **desiccation-related protein PCC13-62 precursor**

Peptide sequences: **R.DVATQFCYQEVGHLR.A; R.VPSYAGGVAEITAR.I**

PFF Mascot score: **[93]**   Sequence coverage %: **[9]**

Matched peptides No.: **[2]**   p value: **0.02**

Calculated Mr:**34238**   Calculated pI: **4.82**

PFF Searched Score:

Ions score is -10*Log(P), where P is the probability that the observed match is a random event.
Individual ions scores > 51 indicate identity or extensive homology (p<0.05).
Protein scores are derived from ions scores as a non-probabilistic basis for ranking protein hits.

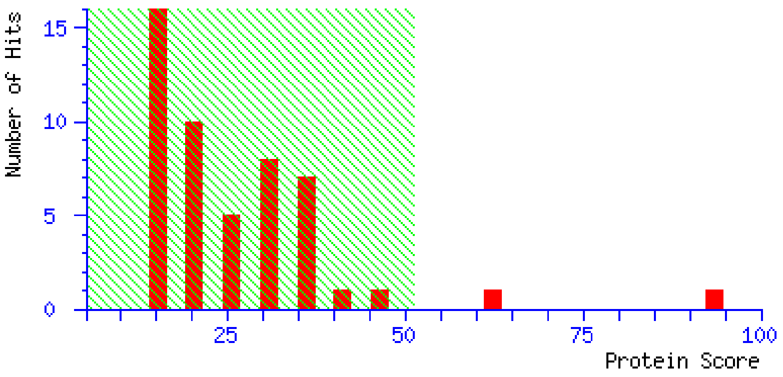

Matched peptide sequences shown in Bold Red

1 MGAPALSSVV VVATAAVVCL CGAFAQAQDM DSEWARPAYR GFFGAPAGSL
51 LPQSDVDLLE FPLNLEYLET EFFCWSALGY GLDAIDANLT GGGPPSIGGQ
101 SASLTPFIR**D** **VATQFCYQEV** **GHLR**AIKQTV RGFPRPLLDI SAANFGKIIE
151 QALNATLDPP FDPYENSLNF LVASYIIPYV GLTGYVGANP RLLTPQARKL
201 LAGLLAVESA QDAVIRTLLY ERGMAR**VPSY** **AGGVAEITAR** ISDLRNSLGR
251 RGVKDEGLVV APELGPEGLT VGNIIAGDHL SLAYDRTPEE ILGIVYGTGN
301 SAQHGGFFPQ GADGRIARGL LA

Spot No.: **22**

NCBI accession No: **gi|226493460**

Plant species: **Zea mays**

Protein name：**stem-specific protein TSJT1**

Peptide sequences: **K.DEVFCLFEGVLDNLGR.L; R.SFAAKDEVFCLFEGVLDNLGR.L**

PFF Mascot score: **[125]** Sequence coverage %: **[8]**

Matched peptides No.: **[2]** p value: **5.0e-05**

Calculated Mr:**25050** Calculated pI: **5.23**

PFF Searched Score:

Ions score is -10*Log(P), where P is the probability that the observed match is a random event.
Individual ions scores > 50 indicate identity or extensive homology (p<0.05).
Protein scores are derived from ions scores as a non-probabilistic basis for ranking protein hits.

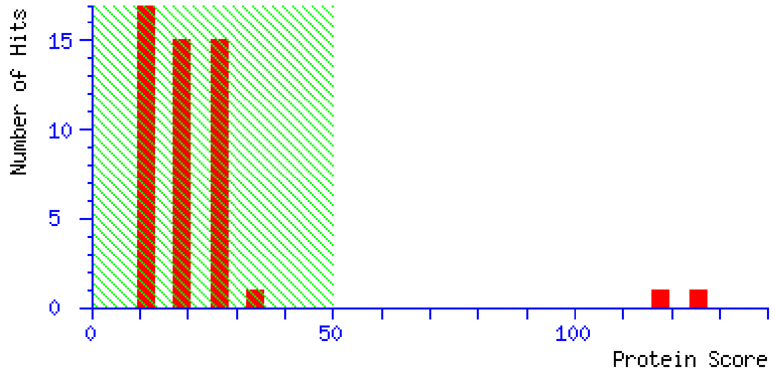

Matched peptide sequences shown in Bold Red

1 MLAVFSGEVV EVPAELVAAG SRTPSPKTKA SELVARFLGT SCPAAVSVRL
51 ADLGHLAYSH ANQALLRPR**S** **FAAKDEVFCL** **FEGVLDNLGR** LSQQYGLSKG
101 ANEVLLVIEA YKALRDRAPY PASLMLAQLA GAYAFVLFDA STNSLLVASG
151 GDVPLFWGVT ADGCVAFSDD IDVLKGSCGK SLAPFPQGCF YSNALGGLKC
201 YENPKNKVTA VPANEEEICG ATFQVEGATV LTALH

Spot No.: **23**

NCBI accession No: **gi|226499536**

Plant species: ***Zea mays***

Protein name: **NADH-ubiquinone oxidoreductase 23 kDa subunit**

Peptide sequences: **K.DWNAVFER.S; R.SINTLFLTEMVR.G; K.LCEAICPAQAITIEAEER.E**

PFF Mascot score: **[210]** Sequence coverage %: **[17]**

Matched peptides No.: **[3]** p value: **5.8e-08**

Calculated Mr:**26093** Calculated pI: **5.24**

PFF Searched Score:

Ions score is -10*Log(P), where P is the probability that the observed match is a random event.
Individual ions scores > 51 indicate identity or extensive homology (p<0.05).
Protein scores are derived from ions scores as a non-probabilistic basis for ranking protein hits.

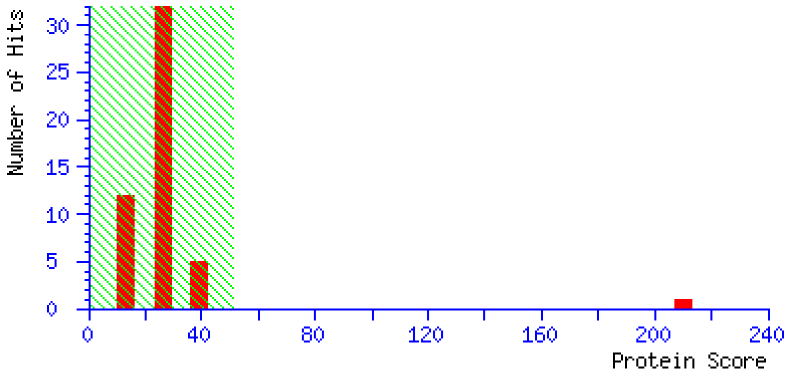

Matched peptide sequences shown in Bold Red

```
  1 MASLLARQAA QALRARQTAQ LGPAASAMQG HLRTYMNAGI PKRFKEDEEK
 51 EQLAKDVAKD WNAVFERSIN TLFLTEMVRG LMLTLKYFFD RNVTINYPFE
101 KGPLSPRFRG EHALRRYESG EERCIACKLC EAICPAQAIT IEAEEREDGS
151 RRTTRYDIDM TKCIYCGFCQ EACPVDAIVE GPNFEFATET HEELLYDKEK
201 LLENGDRWET EIAENLRSES LYR
```

Spot No.: **24**

NCBI accession No: **gi|32330695**

Plant species: **Zea mays**

Protein name： **SKP1/ASK1-like protein**

Peptide sequences: **K.SSDGEEFEVEEAVAMESQTIR.H; K.NDFTPEEEEEIR.R; K.NDFTPEEEEEIRR.E**

PFF Mascot score: **[301]**   Sequence coverage %: **[19]**

Matched peptides No.: **[3]**   p value: **4e-12**

Calculated Mr: **19202**   Calculated pI: **4.48**

PFF Searched Score:

Ions score is -10*Log(P), where P is the probability that the observed match is a random event.
Individual ions scores > 50 indicate identity or extensive homology (p<0.05).
Protein scores are derived from ions scores as a non-probabilistic basis for ranking protein hits.

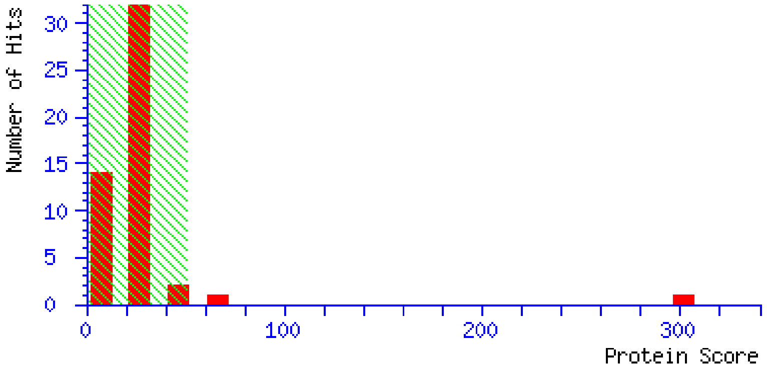

Matched peptide sequences shown in Bold Red

```
  1 MAAAGDAGEK KMITLKSSDG EEFEVEEAVA MESQTIRHMI EDDCADNGIP
 51 LPNVNSKILS KVIEYCNKHV QAKPADGAAA GAGAGASDAA PAAPAEDLKN
101 WDAEFVKVDQ ATLFDLILAA NYLNIKGLPD LTCQTVADMI KGKTPEEIRK
151 TFNIKNDFTP EEEEEIRREN QWAFE
```

Spot No.: **25**

NCBI accession No: **gi|195636212**

Plant species: ***Zea mays***

Protein name: **rhicadhesin receptor precursor**

Peptide sequences: **R.NPGNTNNPAGSVVTAANVEK.F; K.FPGVNTLGVSMAR.I; R.IDYAPGGQNPPHTHPR.A; K.GDVFVFPR.G; R.GLVHFQQNR.G**

PFF Mascot score: **[385]** Sequence coverage %: **[30]**

Matched peptides No.: **[5]** p value: **2.2e-08**

Calculated Mr:**22948** Calculated pI: **6.58**

PFF Searched Score:

Ions score is -10*Log(P), where P is the probability that the observed match is a random event.
Individual ions scores > 50 indicate identity or extensive homology (p<0.05).
Protein scores are derived from ions scores as a non-probabilistic basis for ranking protein hits.

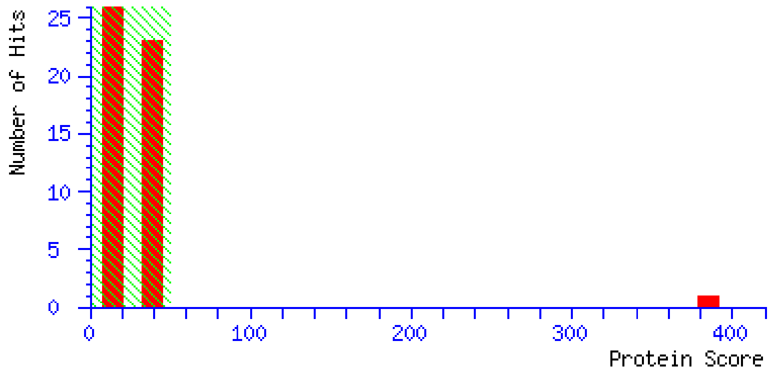

Matched peptide sequences shown in Bold Red

```
  1 MAKVHLYVAA ACAVVLALAA PALAGDPDML QDVCPADYAS PVKLNGFACK
 51 ANFSADDFFF DGLRNPGNTN NPAGSVVTAA NVEKFPGVNT LGVSMARIDY
101 APGGQNPPHT HPRATEIIFV LEGTLEVGFI TTANALFTKT VTKGDVFVFP
151 RGLVHFQQNR GHGPAAVVAA FNSQLQGTQA IAMTLFGAVP PVPSDILAKA
201 FRISSGEVDH IKANFAPK
```

Spot No.: **26**

NCBI accession No: **gi|195640298**

Plant species: **Zea mays**

Protein name: **glycine-rich RNA-binding protein 7**

Peptide sequences: **K.LFVGGLSYGTDDHSLR.D; K.LFVGGLSYGTDDHSLRDEFAK.Y**

PFF Mascot score: **[86]** Sequence coverage %: **[8]**

Matched peptides No.: **[2]** p value: **0.068**

Calculated Mr:**25155** Calculated pI: **4.87**

PFF Searched Score:

Ions score is -10*Log(P), where P is the probability that the observed match is a random event.
Individual ions scores > 51 indicate identity or extensive homology (p<0.05).
Protein scores are derived from ions scores as a non-probabilistic basis for ranking protein hits.

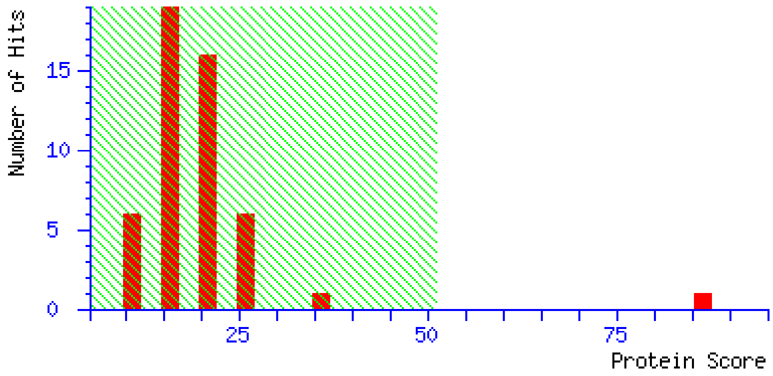

Matched peptide sequences shown in Bold Red

```
  1 MMLANKLGGL LKNATSSKSS NMSVYQAIRC MSSSKLFVGG LSYGTDDHSL
 51 RDEFAKYGQV IEAKIILDRE SGRSRGFGFI TYTSSEEASA AITAMDGKTL
101 DGRNIRVNHA NERTGGFRSS GGGYGGGGYG GGYGGGSGGY GGGGSGDYGG
151 GSGGYGGNYG NRAGGGYGGG GDYGVAGGAE GSFAAGGSDS FGSSNFADSG
201 FGENPAGSFG ATGGSSGADG FSAGTPGDGN KNDEIMDDLF KDDEPDSYAN
251 KRS
```

Spot No.: **27**

NCBI accession No: **gi|162457809**

Plant species: **Zea mays**

Protein name：**ubiquitin-conjugating enzyme protein E2**

Peptide sequences: **R.FTSEMWHPNVYPDGR.V; R.VCISILHPPGEDPNGYELASER.W**

PFF Mascot score: **[85]**   Sequence coverage %: **[21]**

Matched peptides No.: **[2]**   p value: **0.12**

Calculated Mr: **19072**   Calculated pI: **5.04**

PFF Searched Score:

Ions score is -10*Log(P), where P is the probability that the observed match is a random event.
Individual ions scores > 50 indicate identity or extensive homology (p<0.05).
Protein scores are derived from ions scores as a non-probabilistic basis for ranking protein hits.

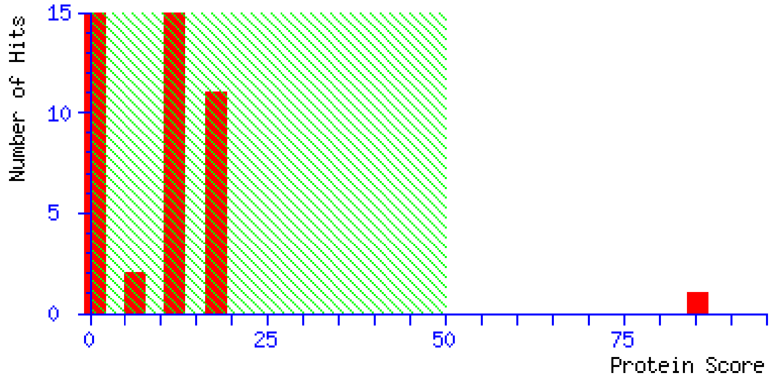

Matched peptide sequences shown in Bold Red

```
  1 MASSASQASL LLQKQLKDLA KNPVDGFSAG LVDDSNIFEW QVTIIGPPDT
 51 LYDGGYFNAI MTFPQNYPNS PPSVRFTSEM WHPNVYPDGR VCISILHPPG
101 EDPNGYELAS ERWTPVHTVE SIVLSIISML SSPNDESPAN IEAAKDWREK
151 RDEFKKKVRQ CVRKSQEML
```

Spot No.: **28**

NCBI accession No: **gi|238009876**

Plant species: **Zea mays**

Protein name: **protease PrlC candidate 1**

Peptide sequences: **K.SPLEVFVSFR.G; K.EAVLSGVALEDEQR.E**

PFF Mascot score: **[81]** Sequence coverage %: **[3]**

Matched peptides No.: **[2]** p value: **0.34**

Calculated Mr:**88301** Calculated pI: **5.82**

PFF Searched Score:

Ions score is -10*Log(P), where P is the probability that the observed match is a random event.
Individual ions scores > 51 indicate identity or extensive homology (p<0.05).
Protein scores are derived from ions scores as a non-probabilistic basis for ranking protein hits.

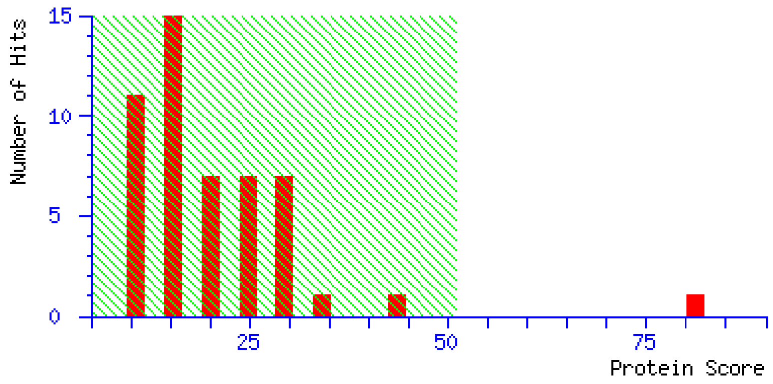

Matched peptide sequences shown in Bold Red

```
  1 MGPPRVGVGV GVGGGSGIGV AMVFLIVSAS SFLIRLPLVA RARLPNPTSS
 51 SYTSRSRSRA LLLLPASSPL RAFCPASRRP SPATCSAAYA SSSMATDDNP
101 LLADFDFPPF DRVEPIHVRP AVRTLLARLE GELTDLEKGV QPTWGKLVEP
151 LERIVDSLEV VWGTVDHLKA VKDSSDLRAA VEDVQPDKVK FQLRLGQSKP
201 IYQAFNAIRN SSDWETLSEA RKRIVEAQIK EAVLSGVALE DEQREKFNQI
251 EQELEKLTQK FSENVLDATK KFEKLITDKN EIDGLPATAL GLAAQTAASK
301 GHENASAENG PWIITLDAPS YIAVMQHARN RALREEVYRA YLTRASSGDL
351 DNTNIISQIL KLRLEKAKLL GYKNYAEVSM AQKMATVDRV EELLEKLRAA
401 SWDHAVKDME DLKAFAKESA SPEANDLAHW DLSFWSERLR ESKYDINEED
451 LRPYFALPKV MDGLFSLANR LFGVSVEPAD GLAPVWNSDV KFYCVKDSSN
501 SPVAYFYFDP YSRPSEKRGG AWMNVVFSRS RVLARNGSPV RFPVAHMVCN
551 QTPPVGDKPS LMTFREVETV FHEFGHALQH MLTKQDEGFV SGIRGVEWDA
601 VELPSQFMEN WCYHKNTLLS IAKHYETGEL LPEEIYAKLV AAKNFRAGTF
651 SLRQIRFASV DMELHTTYDP NGSLSIYDVD RRVAERTQVL APLPEDKFLC
701 SFSHIFAGGY AAGYYSYKWA EVLSADAFSA FEDVGLDNEK AIEETGRRFR
751 ETVLALGGGK SPLEVFVSFR GREPSPEALL RHNGLLPVAA L
```

Spot No.: **29**

NCBI accession No: **gi|413949328**

Plant species: ***Zea mays***

Protein name： **pyruvate, phosphate dikinase 3, Precursor**

Peptide sequences: **R.FAYDSFR.R; K.VLANADTPEDALAAR.N; R.SDFEGIFR.A; R.LLDPPLHEFLPEGNVEEIVR.E**

PFF Mascot score: **[103]** Sequence coverage %: **[5]**

Matched peptides No.: **[4]** p value: **1.7**

Calculated Mr: **103777** Calculated pI: **6.04**

PFF Searched Score:

Ions score is -10*Log(P), where P is the probability that the observed match is a random event.
Individual ions scores > 49 indicate identity or extensive homology (p<0.05).
Protein scores are derived from ions scores as a non-probabilistic basis for ranking protein hits.

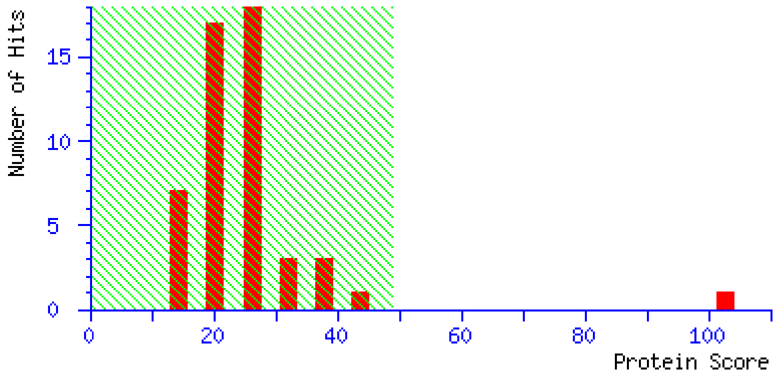

Matched peptide sequences shown in Bold Red

```
  1 MAASVSTSRV ICLQKPGPEG SSRGAREVAP FTRQSVAAPR RPHGANASVI
 51 IGSDSGAGRG QHSPPLRAVV DAGPVQTTKK RVFHFGKGKS EGNKNMKELL
101 GGKGANLAEM ASIGLSVPPG FTVSTEACQQ YQEAGRALPP GLWAEVLDGL
151 RWVEEYMGAA LGDPRRPLLL SVRSGAAVSM PGMMDTVLNL GLNDQVAAGL
201 AAKSGDRFAY DSFRRFLDMF GNVVMDIPHA LFEEKLEAMK KAKGLKNDTD
251 LTATDLKELV SQYKNVYVEA KGEPFPSDPK RQLELAVLAV FDSWESPRAK
301 KYRSINQITG LRGTAVNVQC MVFGNMGNTS GTGVLFTRNP NTGEKKLYGE
351 FLVNAQGEDV VAGIRTPEDL DAMKDVMPQA YKELVENCRI LESHYKEMQD
401 IEFTVQESRL WMLQCRTGKR TGKSAVKIAV DMVNEGLVER RAAIKMVEPG
451 HLDQLLHPQF ENPSAYKDQV IATGLPASPG AAVGQVVFTA EDAETWHSQG
501 KSVILVRAET SPEDVGGMHA AAGILTERGG MTSHAAVVAR GWGKCCVSGC
551 SGIRVNDAEK VVKIGGNVLR EGEWLSLNGS TGEVILGKQP LSPPALSGDL
601 GTFMSWVDDV RKLKVLANAD TPEDALAARN NGAEGIGLCR TEHMFFASDE
651 RIKAVRQMIM APTVELRQQA LDRLLPYQRS DFEGIFRAMD GLSVTIRLLD
701 PPLHEFLPEG NVEEIVRELC SETGANQEDA LARIEKLSEV NPMLGFRGCR
751 LGISYPELTE MQARAIFEAA IAMTNQGVQV FPEIMVPLVG TPQELGHQVA
801 LIRQVANKVF TSMGKTIGYK IGTMIEIPRA ALVADEIAEQ AEFFSFGTND
851 LTQMTFGYSR DDVGKFIPIY LAQGILQHDP FEVLDQRGVG ELVKLATERG
901 RKARPNLKVG ICGEHGGEPS SVAFFAKTGL DYVSCSPFRV PIARLAAAQV
951 LV
```

Spot No.: **30**

NCBI accession No: **gi|48374986**

Plant species: **Zea mays**

Protein name: **hypothetical protein Z477F24.14**

Peptide sequences: **R.LAAFYEAVLGFER.I**

PFF Mascot score: **[66]** Sequence coverage %: **[9]**

Matched peptides No.: **[1]** p value: **0.0014**

Calculated Mr: **15597** Calculated pI: **4.94**

PFF Searched Score:

Ions score is -10*Log(P), where P is the probability that the observed match is a random event.
Individual ions scores > 51 indicate identity or extensive homology ($p<0.05$).
Protein scores are derived from ions scores as a non-probabilistic basis for ranking protein hits.

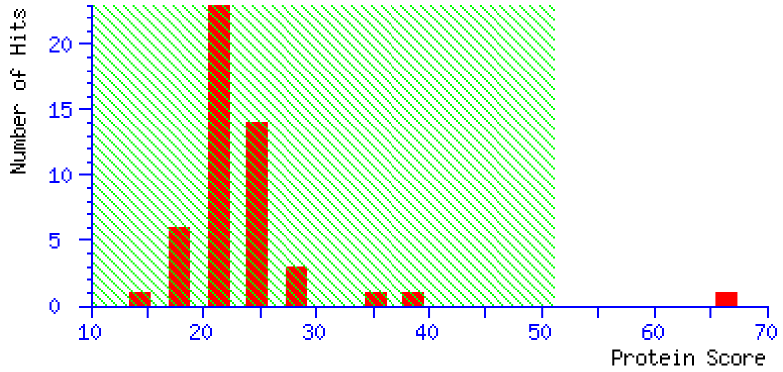

Matched peptide sequences shown in Bold Red

```
  1  MATLQLNHIA RETSDVARLA AFYEAVLGFE RIPSPTYSGF QVAWLRLPSS
 51  PDVALHLIER DPAAAPVAVG PGAEGAPPSQ LPRRHHLAFS VADYDGFVTG
101  LKARGTDVFE KSQPDGRTRQ VFFFDPDDEE DLVFTKLGLD VPS
```
